# Supplementary material for: AMPA receptor GluA2 subunit defects are a cause of neurodevelopmental disorders
Source: Nat Commun. 2019 Jul 12;10:3094. doi: 10.1038/s41467-019-10910-w (PMC6626132; doi:10.1038/s41467-019-10910-w)
Supplement: Supplementary file 2 — Supplementary Information [file 41467_2019_10910_MOESM2_ESM.pdf]

AMPA receptor GluA2 subunit defects are a cause of neurodevelopmental disorders

Salpietro et al.

Supplementary Note 1

This 3.5-year-old Spanish boy was born at term after an uneventful pregnancy. His length and weight at birth were within normal values and his occipital-frontal circumference (OFC) was 35 cm (25th centile). He had episodes of focal tonic and hypomotor seizures from birth. Electroencephalographic (EEG) recordings showed multifocal spikes and sharp waves during the neonatal period (Supplementary Table 5). Follow-up video-EEG recording of the patient showed high-voltage delta activity and superimposed sharp wave-slow wave complexes over the anterior cerebral regions followed by left eye deviation and tonic posturing of the upper limb associated with right frontal slow wave-spike wave complex (Supplementary Video 1) He failed to reach any developmental milestones and never acquired the ability to walk. He had slow head growth and at the age of 2 years and 11 months his OFC was 44.5 cm (<3rd centile). At 3 years, he showed severe axial hypotonia and hypokinesia (Supplementary Video 2) with no visual interaction. Intermittent strabismus and oculogyric crisis were also noted (Supplementary Video 5). Ictal video-EEG recording captured a focal seizure characterized by staring and oral automatisms associated with high-voltage delta activity and superimposed sharp wave-slow wave complexes over the anterior cerebral regions, followed by left eye deviation and tonic posturing of the upper limb, associated with right frontal slow wave-spike wave complex. The seizure rapidly proceeded to a clonic phase lasting several minutes (Supplementary video 1). At the current age of 3.5 years seizures occur on a daily basis. Serial brain MRI scans show signal abnormalities suggestive of hypo-myelinating leukodystrophy (Figure 2b), with global reduction in the cerebral white matter volume and cerebellar atrophy with prominent vermian involvement. Several antiepileptic drugs (AEDs) were trialed, including pyridoxine, vigabatrin, valproic acid, levetiracetam, clobazam, and topiramate, without significant clinical response, as well as a ketogenic diet. Trio whole exome sequencing (WES) identified a *de novo* G>T transversion at chromosome 4:158282233 (GRCh37) for which the NM001083619.1 transcript (NM001083619.1: c.2363G>T, p.Trp788Leu) was used, as this position lies in an alternatively spliced exon that is not present in NM000826.3. A *de-novo* truncating variant of uncertain significance was also identified in this Patient by trio WES (NM_018017:exon5:c.1072_1073del:p.G358Tfs*6) in the *CCDC186* gene.

Supplementary Note 2

Patient 2 is a 13-year-old British girl with NDD including moderate ID and ASD, previously recruited within the DDD Study in the UK (https://www.ddduk.org/). She was born at term with an OFC of 33.5 cm (10th- 25th centile). OFC at 9 months was 43.5 cm (25th centile) and at the age of 10 years and 11 months was 50.5cm (< 0.4^{th}). She was slightly delayed in her development. Visual interaction and social communication (including smiling) during the first year of life were normal. During the second year of life, abnormal behavior was noted with frequent shouting and diminished social interaction and communication. She was diagnosed with ID and autistic and RTT-like features (Supplementary Table 6) in childhood. No seizures were noted, and EEG studies were reported as normal. Brain imaging studies were reported as normal. At the current stage, she has mild dyspraxic gait and makes eye contact. Behavioral abnormalities include episodes during which is agitated as well as episodes of self-harm. She has frequent screaming spells, often associated with breath-holding and stereotypies, including hand-wringing and clapping (Supplementary Video 3). Her speech is severely delayed and only 4-5 words are currently intelligible. At the last clinical evaluation at the age of 13 years OFC was 51.5 cm (10th-25th centile). Microarray analysis was

normal. RTT was suspected but *MeCP2* testing was negative. WES revealed a *de novo* in frame deletion in *GRIA2* (NM000826.3: c.1582_1590del; p.Pro528_Lys530del).

Supplementary Note 3

Patient 3 is a 19-year-old Caucasian boy who presented in early infancy with mild developmental delay with abnormal behavior. As a child he frequently experienced episodes of aggressive behavior with self-harm and screaming spells and was diagnosed with ID and ASD. He never had seizures and EEG was normal. MRI did not show any abnormality. At the current age of 19 years, he exhibits head banging and self-injurious behaviors (Supplementary Video 4). During childhood he acquired the ability to formulate up to 40 words and short sentences; however, during late childhood developmental regression became evident and he became non-verbal around the age of 10. Stereotypies are present (Supplementary Table 6). On neurological examination he presents some stiffness. As part of his behavioral phenotype, some obsessive-compulsive traits and repetitive behaviors are also evident. WES revealed a *de novo* missense variant in *GRIA2* (NM000826.3: c.1831G>A; p.Asp611Asn).

Supplementary Note 4

Patient 4 is a 19-year old British girl born at term after an uneventful pregnancy. Her growth parameters at birth were within normal range and her OFC was at the 25th-50th centile. She has a history of hypotonia and developmental delay (including delay in sitting and crawling) from infancy. During childhood, behavioral abnormalities including episodes of agitation and self-harm became evident. She has frequent RTT-like stereotypies (Supplementary Table 6) including hand-wringing, repeated clapping, rocking and raspberry blowing. She has abnormalities of the sleep-wake cycle, with frequent desire for periods of prolonged sleeping, which have increased over time. She has an ataxic gait, tremor, and dystonic attacks which are triggered by unexpected noises. She was suspected to have absence seizures at the age of 3 years but has had no generalized seizures and EEG was normal at that age. She has an history of developmental regression. Currently, she has severe speech impairment (aphasia). Her MRI scan (not shown) at the age of 3 years showed delayed myelination and undefined periventricular white matter changes but the brain including the cerebellum is otherwise normal. Trio WES identified a *de novo* missense variant in *GRIA2* (NM000826.3: c.1825G>A; p.Gly609Arg).

Supplementary Note 5

Patient 5 is a 10-year-old Turkish boy with mild developmental delay and intellectual disability, some autistic features and speech delay. On clinical examination at the age of 5 years his OFC was within normal ranges. He had one febrile convulsion at the age of 12 months and was briefly treated with valproic acid with no further seizures. At that time EEG showed some non-specific left centro-temporal features, but follow-up EEG was normal. WES identified a *de novo* missense variant in *GRIA2* (NM_000826.3: c.905A>G; Asp302Gly)

Supplementary Note 6

Patient 6 is a 31-year-old Caucasian female with a history of developmental delay since infancy. She sat unsupported at nine months, crawled at 15 months, and walked independently at 20 months. Currently, she exhibits tip-toe gait. Her speech was severely delayed, and only a few short sentences

are intelligible. There is no evidence of developmental regression (Supplementary Table 6). She carries a diagnosis of intellectual disability (IQ of 40 to 50, although this may be an underestimate given she has substantially better receptive than expressive language function). She has an autism spectrum disorder (ASD) with some atypical features, attention deficit hyperactivity disorder (ADHD) and dyspraxia. She also exhibited behavioural abnormalities from early infancy. As an infant, she was very irritable and would cry often. She also had poor eye contact. She exhibited head banging and self-injurious behaviours as a child. She continues to exhibit episodic aggression toward others, albeit infrequently. She also has tactile sensitivity around her face and mouth (does not like people brushing her teeth). A distinct feature of her current mental status and behaviour is that, although she is highly perseverative, she sustains remarkably good eye contact, and is friendly, especially with new people, and exhibits an unusually high level of "eagerness" bordering on overt psychomotor agitation whenever she successfully shares an idea or interest (vigorously nodding, smiling, repeating, tensing her entire body, and displaying intense affective arousal). There is no history of seizures. Physical examination at the age of 29 years revealed a small body size (height ~25th centile and weight ~3rd centile), and microcephaly (OFC 52.5 cm, <3rd centile). She had normal brain imaging. She had a normal array- comparative genome hybridization (CGH) and unremarkable metabolic work-up. WES revealed a *de novo* missense variant in *GRIA2* (NM000826.3: c.2700G>T; p.Gly792Val). A *de-novo* variant of uncertain significance was also identified in this Patient by trio WES (NM_001127235: c.212dupA; p.Asn71Lysfs*29) in the *GPBP1* gene (MIM #608412).

Supplementary Note 7

Patient 7 is an 11-year-old Latino-American girl who started with daily clusters of infantile spasms since the age of 15 months. Additional seizure types included atypical absences and nocturnal episodes characterized by upper limb stiffening in extension and breathing changes. EEG showed diffuse polyspike and slow spike and wave at 2.5 C/Sec. She has received multiple AEDs including clonazepam clobazam, felbamate, rufinamide, levetiracetam, valproic acid, lamotrigine and perampanel, but clinical response was poor, and ketogenic diet also failed to bring about an improvement (Supplementary Table 5). She also showed global developmental delay with severe ID, abnormal behavior with RTT-like features (including head nodding, rocking and breathing with frequent hyperventilation episodes, Supplementary Table 6 and Supplementary Video 5). At the age of 10 years her height was 134 cm (25th- 50th centile) and weight 26 kg (10th centile). She never acquired the ability to walk. RTT was considered and she was negative for *MeCP2* variants. Large epilepsy multigene panels failed to identify a mutation. Trio WES identified a *de novo* missense variant in *GRIA2* affecting the Q/R RNA editing site (NM000826.3: c.1819C>G; p.Gln607Glu/p.Arg607Gly). Sequencing data were reviewed for evidence of mosaicism in both the Proband and parent samples as the *de-novo* variant affecting the Q/R site was present only in 14% of exome sequencing reads and present at an allele ratio of < 35% in the Proband using both NGS and ABI data. WES also identified the de novo variant of uncertain significance (NM_003870: c.4186A>G; p. I1396V) in the gene *IQGAP1* (MIM #603379).

Supplementary Note 8

Patient 8 is a 2-year-old boy of mixed Caucasian and Latino American ancestry. He was born preterm (at 30 weeks of gestation). He has an history of delay of his developmental milestones. WES revealed a *de novo* variant in *GRIA2* (NM000826.3: c.88+2T>C; p.?) variant which is likely to affect

splicing of exon 1. This variant is predicted by three in silico tools (MaxEnt, NNSPLICE, HSF) to result in an abolishment of the natural donor side and strengthening of an alternative donor splice site at position c.88+4 (Supplementary Fig. 4).

Supplementary Note 9

This is a 9-year-old boy born at term after an uneventful pregnancy. At birth, his length 47 cm and OFC of 33.5 cm (10th centile). He has a history of developmental delay, ID and ASD. He presents joint hyper-mobility. He never had a documented seizure, and his EEG and his brain imaging studies were normal. WES revealed a *de novo* missense variant in *GRIA2* (NM000826.3: c.1582C>A; Pro528Thr).

Supplementary Note 10

This is a 5-year-old Chinese girl with ID and ASD. At her current age height is 111cm, weight is 19kg and OFC is 49.5cm (25th-50th centile). The first abnormalities were noted in infancy with impaired social interaction and abnormal behavior. The patient was diagnosed with severe ID (PPTV at 4.5 years: 57). There is also a history of ritualistic behavior and unusual preoccupations such as focusing at line objects and neon lamp. At her current age she presents speech delay (first words at the age of 16 months) and cannot complete intelligible sentences. She has never had seizures. She has autistic features and obsessive-compulsive thoughts and behaviors. Targeted Capture and High-Throughput Sequencing Using Molecular Inversion Probes (MIPs) identified a *de novo* frameshift deletion in *GRIA2* (NM000826.3:c.1785del; p.Phe595LeufsX37), predicted to introduce a premature truncation in the resulting GluA2 protein.

Supplementary Note 11

Patient 11 is an 8-year-old Chinese Han boy affected with severe ID and ASD. At birth, his growth parameters were reported within normal ranges (25th- 50th). At the current age of 8 years OFC is 48.5 cm (10th centile). The first abnormalities were seen at 17 months of age with impairment of communication and social interaction. The patient was diagnosed with severe ID (Gesell at 3 years: adaptive: 58, gross motor: 83, find motor: 60, language: 58, personal-social behavior: 66). There was also a history of speech delay with the first words pronounced at the age of 19 months. At his current age he still cannot complete phrases. Brain imaging was unremarkable. Seizures have not been noted. The behavior is characterized by hyperactivity and autistic features, including ritualistic behaviors. In this Patient, targeted sequencing with MIPs identified a *de novo* , a *GRIA2 de-novo* variant (NM000826.3:c.1844+1G>A; p.?) which is predicted to cause a complete loss of donor splice site of exon 11 according to all tools for which predictions were available (Supplementary Fig. 5).

Supplementary Note 12

Patient 12 is a 6-year-old boy with a history of mild developmental delay, ID and ASD. At his current age of 6 years his height is 110 cm, weight is 20kg and OFC is 50 cm (25th – 50th centile). The first abnormalities were noticed during late infancy around the age of 2 years, when a delay in speech and cognition were noted. At the age of 3.4 years ID was documented (Gesell at 3.4 years: 37). Speech was severely impaired with the first words pronounced at the age of 2 years. At his present age, he still cannot communicate or pronounce phrases. His behavior is characterized by hyperactivity and autistic features. He presents obsessionality such as a special interest in whirligigs.

Targeted sequencing with MIPs identified a *de novo* frameshift deletion (NM000826.3:c.857del; p.Pro286LeufsX14) in *GRIA2*.

Supplementary Note 13

Patient 13 is a 3-year-old girl born preterm at 30 weeks of gestation, because of maternal pre-eclampsia, by normal delivery. At birth, her OFC was 33 cm (15th centile). She had postnatal deceleration of head growth and at the age of 2 years and 9 months OFC was 42.5 cm (<3rd centile). During the second month of life, she started experiencing focal myoclonic seizures, with eyelid myoclonus and chewing. EEG showed prominent spike and slow spikes over the right centro-temporal region. At this stage, myoclonic and clonic seizures occurred between 15 and 40 times per day. She never reached any motor milestone or made visual contact. She also had feeding difficulties. Several antiepileptic treatments, including clonazepam, topiramate, phenobarbital, and ketogenic diet failed to improve her epilepsy. Since the second year of life, asymmetrical dystonia and dyskinesia were also noted. Neurological examination at the age of 2 years revealed severe hypotonia associated with spasticity. Brain imaging showed generalized reduction in the cerebral white matter volume and cerebellar atrophy with vermian deficiency (Fig. 2 d-f). WES revealed a *de novo* missense variant in *GRIA2* (NM000826.3: c.1937C>A; p.Thr646Asn).

Supplementary Note 14

Patient 14 is an 8-year-old girl from Korea. Her first symptoms started during early infancy with severe hypotonia and developmental delay. She has severe ID. Her motor milestones were delayed and she never walked although she is able to sit without support. She presented autistic features and aggressive behaviour since early childhood. She also has emotional lability with inappropriate screaming or crying. She has no purposeful movements of the hands. She also has a history of difficulty falling sleep. Her speech impairment is severe, and she never pronounced intelligible words. Trio WES identified a *de novo* missense variant in *GRIA2* (NM000826.3: c.1932C>A; p.Phe644Leu).

Supplementary Note 15

Patient 15 is a 13-year-old Chinese Han girl with ASD and ID. There is no history of neurological disorders in the family. During her infancy she was diagnosed with speech and development delay, and abnormal behavior with hyperactivity and autistic features. There is no history of seizures. MIPs identified a *de novo* missense variant in *GRIA2* (NM000826.3:c.140G>A; p.Gly47Glu).

Supplementary Note 16

Patient 16 is a 9-year-old boy with developmental and epileptic encephalopathy. At birth, his OFC was 35 cm (30th centile). During the first week of life, benign neonatal sleep myoclonus was noted. At the age of 3 months episodes of trunk and arms stiffness triggered by intercurrent illness were observed. At the age of 6 months he was diagnosed with epileptic spasms. At this stage, spasms occurred in cluster and with a daily frequency. Other seizure types included tonic and tonic-clonic seizures, status epilepticus and focal impaired awareness seizures (FIAS). At 7 months, video-EEG showed FIAS with either left or right frontal origin.

The patient exhibited profound delay in motor milestones and did not reach the ability to walk. Also there is a history of regression of previously acquired abilities such as smiling, rolling and grasping. He is able to make visual contact and to follow. Several antiepileptic treatments including phenobarbitone, phenytoin, popiramate, clonazepam, levetiracetam, valproic acid and oxcarbazepine, failed to control his epilepsy, although an improvement with ketogenic diet (given with phenytoin, topiramate, phenobarbitone and clonazepam) was observed. Follow-up EEG at the age of 7 years showed bilateral epileptiform activity (left > right) and an abnormally slow background. Episodes of intermittent eye and head deviation to right, intermittent facial jerking, abnormal movements of upper and lower limb without EEG correlate were also noticed. Brain imaging showed a mild progressive cerebral atrophy (brain imaging not shown). Proband-father WES revealed a missense variant in *GRIA2* (NM_000826.3: c.1939G>C; p.Val647Leu) which was confirmed *de novo* through targeted sequencing in the mother. Paternity and maternity were confirmed via targeted sequencing.

Supplementary Note 17

This is a 3-month-old Ashkenazi Jewish boy born at term after an uneventful pregnancy and normal delivery. Birth weight was 3.16 kg and OFC was 34.5 cm (25th centile).

At the age of 3 months OFC was 38.5 cm (10th centile). After birth he had an episode of apnoea associated with low muscle tone and respiratory insufficiency. He was then intubated and phenobarbital treatment was given because of suspected seizures. At day 4 of life his neurological examination revealed myoclonus in the face and limbs. However, at that time the EEG showed normal background activity with no epileptic activity. He was started on clonazepam in addition to the phenobarbital. At day 7 of life he started suffering from focal seizures characterized by eye blinking and mastication, sometimes associated with hypertonia and bradycardia. Episodes of increased tone, bradycardia, and breathing abnormalities (with short of apnoea) were observed in the first weeks of life. An exaggerated startle response was noted. EEG showed bilateral, mainly temporal, non-synchronized epileptic activity with multiple focal ictal electrographic events. A metabolic and genetic work-up was then performed and reported as normal. Magnetic resonance imaging (MRI) of the brain revealed cerebellar vermis hypoplasia, with otherwise normal brain structure (Fig. 2 g-i). Several AEDs, including phenytoin, carbamazepine, clonazepam, topiramate, levetiracetam, valproic acid, pyridoxine, pyridoxal 5-phosphate, vigabatrin, ketamine, lacosamide, and perampanel failed to control his seizures. At age 2.5 months, he was on ketogenic diet, phenobarbital, clonazepam, and topiramate. Clinical examination demonstrated microcephaly (OFC 38.5 cm, < 5th) and marked (especially axial) hypotonia. He was noted to have some visual contact but did not reach any motor or developmental milestones. At the age of 3 months he expired (probable SUDEP). Post-mortem trio WES identified a *de novo* missense variant in *GRIA2* (NM_000826.3: c.1915G>T; P.Ala639Ser).

Supplementary Note 18

Patient 17 is a 5-year-old Caucasian boy born at term after uneventful pregnancy and delivery by caesarean section. Weight and height at birth were within normal ranges and OFC was 35 cm (25th centile). On day 4 of life he had episodes of clonus of the right upper limb with post-ictal hypotonia. At that time EEG and brain imaging were reported as normal. During the first 3 months of life, he presented focal motor seizures involving the right face and upper limb associated with brief (several seconds) loss of consciousness. Subsequently in his infancy and childhood the

seizures evolved to a pattern characterized either by fixed gaze with upper limb extensor posturing and breathing changes or, alternatively, frequent tonic attacks (every 2-3 days) characterized by extension of all four limbs associated with staring and abnormal breathing and sialorrhea. At his last clinical examination at the age of 5 years, he has choreo-athetoid movements and lower limbs spasticity. Intermittent strabismus and some abnormal ocular movements are also noted. The head and neck control are normal. Visual interaction is inconstant and brief. The frequency of seizures varies between daily and weekly, with left upper limb and right face clonus. Brain imaging (Fig. 2 j-l) shows global cerebral atrophy with vermian atrophy and white matter changes. EEG shows very slow rhythms with frontal-central delta activity and left frontal discharges. At his current age of 5 years his OFC is 49 cm (15th centile). Several antiepileptic medications have been trialled including phenobarbital, phenytoin, vigabatrin, midazolam, and valproic acid with poor results. Epilepsy improved slightly after the introduction of clonazepam and levetiracetam. He started vocalization although cannot say any intelligible word. Trio WES identified a *de novo GRIA2* variant (NM_000826.3: c.1939G>C; p.Val647Leu).

Supplementary Note 19
This patient is a 5-year-old Chinese Han boy with ASD and ID. There is no history of neurological disorders in the family. During his infancy he was diagnosed with speech delay and delay in development milestones. He has intellectual disability (at the age of 4.5 years non-verbal intellectual quotient: 74; verbal intellectual quotient: 88) and, since the third year of life, abnormal behavior with autistic features and obsessive-compulsive traits was noted. He exhibit hand and finger mannerisms. There is no history of seizures. MIPs identified a *de novo* stop-gain variant in *GRIA2* (NM000826.3: c.967C>T; p.Arg323ter).

Supplementary Note 20
This is a 5-month-old Brazilian female, born by cesarean section after uneventful pregnancy. At birth, she had Apgar score of 9, weight of 3090 g and OFC of 35cm (50-75th). Since the first day of life she developed focal erratic seizures with facial grimacing and abnormal ocular movements. She also had frequent episodes of brief clonic seizures associated with associated cyanosis and respiratory difficulties. The girl remained most of her life artificially ventilated, initially with orotracheal tube and lately with tracheostomy. She also had feeding difficulties and required nasogastric tube and then gastrostomy at 2 months of age. Several antiepileptic trials were trialled including phenobarbital, valproic acid, topiramate, vigabatrin, levetiracetam pyridoxine and pyrodoxal phosphate failed to control her seizures. She did not reach any motor or developmental milestones. Brain MRI imaging showed a marked cortico-subcortical and cerebellar atrophy at the age of 2 months (not shown). Several EEG were performed, and these showed heterogeneous abnormal patterns including burst-suppression like pattern at seizures onset and multifocal spikes (more intense in temporal regions) on follow-up EEGs. At the age of 5 months she expired (probable SUDEP). Post-mortem trio WES analysis identified a *de novo* missense variant in *GRIA2* (NM_000826.3: c.1915G>T; P.Ala639Ser).

Supplementary Note 21
This is a 3-year-old Dutch boy with developmental epileptic encephalopathy. At birth, his OFC was 33 cm (15th). At the age of 2 years and 9 months OFC was 46 cm (<3rd centile). Shortly after birth right sided focal clonic seizures (with associated contralateral deviation of the head) were noticed. Other seizure types observed during the first months of life included tonic clonic, focal, and focal

with secondary generalisation. Seizures usually occurred in cluster of multiple episodes (2-6) a day and were often preceded by staring and blinking with eyes. The boy also had global developmental delay and at the current age he is still unable to sit unsupported. He is non-verbal. Several antiepileptic treatments including phenobarbital, Levetiracetam and valproic acid were trialled with a good clinical response although fever-associated seizures still occur. EEG at onset showed focal discharges over the frontal and parietal areas and an abnormally slow background. Follow-up EEG showed intermittent series of discharges of high amplitude with a maximum over the fronto-central areas (left>right). Brain MRI was reported as normal. Trio WES revealed a *de novo* missense variant in *GRIA2* (NM_000826.3: c.1939G>C; p.Val647Leu).

Supplementary Note 22

Patient 22 is a 3-year-old boy with mild developmental delay and intellectual disability, some autistic features including hand clapping and repetitive behaviours. He has a mixed expressive receptive language disorder with limited functional communication, inability to express needs and echolalia. EEG and brain MRI were normal. WES identified a *de novo* missense variant in *GRIA2* (NM_000826.3: c.2435A>G; p.Asn812Ser).

Supplementary Note 23

Patient 23 is a 30-year-old female with intellectual disability and an history of developmental delay. Since her infancy she had poor eye contact, inappropriate social interaction and repetitive behaviours, which led to a diagnosis of autism spectrum disorder. She has not attained an intelligible speech. She also has echolalia and motor stereotypies. She suffered from insomnia. Her brain MRI was normal. At the age of 8 years she developed a single episode of generalized tonic seizures, at that time valproic acid treatment was started and no further seizure episodes occurred. Trio WES identified a de novo missense variant in *GRIA2* (NM_000826.3: c.2328G>T; p.Glu776Asp).

Supplementary Note 24

Patient 24 is a 5-year-old male with an history of developmental delay. He became able to sit unassisted at the age of 12 months; at the current age of 5 years, he can crawl but cannot walk or stand unassisted. He has not attained any intelligible speech. At the age of 6 months, short-lasting (around 30 seconds) episodes of drooling, lip retraction, and gaze deviation (without post-ictal symptoms) were observed. Sometimes these episodes were longer and progressed to clonic jerking. EEG at onset showed bilateral right frontal epileptic discharges, consistent with a diagnosis of focal seizures. Follow-up EEGs showed multifocal epileptiform abnormalities, on abnormal slowed background. Several antiepileptic drugs were trialled including phenobarbital, levetiracetam, oxcarbazepine and topiramate but these failed to control his seizures. Brain MRI showed a generalized mild atrophy of cerebral parenchymal and white matter loss; also, hippocampal formations appeared to be small (not shown). Trio WES revealed a *de novo* missense variant in *GRIA2* (NM_000826.3: c.1939G>C; p.Val647Leu).

Supplementary Note 25

Patient 25 is a 3 year and 6 months old girl from Canada. Her first symptoms started during early infancy with hypotonia and developmental delay. Her motor milestones were delayed: she sat unassisted at 12 months, crawled at 20 months and walked at the age of 32 months. At 2 months of age she presented with a cluster of apparently generalized tonic–clonic seizures and was put under

valproic acid. At that time, EEG showed multifocal epileptiform activity central-parietal EEG showed multifocal epileptiform activity mainly in the left temporal regions. Since the first year of life autistic features including abnormal behaviour and stereotyped movements (including hand wringing) were noticed. She is non-verbal. At present, her seizures are described as brief focal <1 minute, 1-2 per day with staring, eye deviation and (left) facial twitching. She may also have occasional generalized tonic-clonic seizures. Several antiepileptic treatments including phenobarbitone, phenytoin, clonazepam, levetiracetam, topiramate, valproic acid, oxcarbazepine and cannabinoids failed to control her epilepsy. Trio WES identified a *de novo* missense variant in *GRIA2* (NM000826.3: *:* c.2420C>T; p.A807V).

Supplementary Note 26

This is a 6-year-old British girl with a history of developmental delay since infancy. She started walking at the age of 18 months. She said her first word at the age of 2 years. She has ASD and abnormal behavior. Clinical examination reveals joint hyper-mobility. There is no history of seizures or of any neurological disorders in the family. At the current age of 6 years her height is 101.5cm (25th centile) and OFC is 50.5cm (between 10th and 25th centile). Microarray analysis identified a de novo microdeletion [hg19] 4q32.1 (156,351,739-158,983,901) x1dn encompassing 8 coding genes including *GRIA2.*

Supplementary Note 27

This is a 12-year-old Italian girl with a history of developmental delay since infancy. She started walking at the age of 18 months, and her first words were at 2 years. She was diagnosed with ID, ASD and abnormal behavior. Examination reveals joint hyper-mobility and club feet. There is no history of seizures and EEG at the age of 4 years was reported as normal. Brain imaging was also normal. She has severely impaired speech and currently is non-verbal. Microarray analysis showed a de novo microdeletion [hg19] 4q32.1 (153,038,154-159,949,244)x1dn including *GRIA2.*

Supplementary Note 28

Patient 20 is a 13-year-old Caucasian boy who presented since in early infancy with mild developmental delay and abnormal behavior. At the age of 4 years his OFC was 50.5 cm (25th centile). At the latest cognitive evaluation at the age of 11 years his intellectual quotient (QI) is 74, Quotient Intellectual Verbal (QIV) is 69 and Quotient Intellectual Performance (QIP) is 86. Clinical examination shows poor fine motor skills, impaired speech and abnormal social interaction. Microarray analysis showed a *de novo* microdeletion [hg19] 4q32.1 (157,343,163-158,271,008)x1dn encompassing 3 genes including *GRIA2.*

Supplementary Note 29

Consortia also involved to this study: The Deciphering Developmental Disorders (DDD) Study (http://www.ddduk.org).

Central DDD Team

Wellcome Trust Sanger Institute, Wellcome Trust Genome Campus, Hinxton, Cambridge, CB10 1SA, UK & The Ethox Centre, Nuffield Department of Population Health, University of Oxford, Old Road Campus, Oxford, OX3 7LF, UK

DDD Management Team (*Principal Investigator)
Jeffrey C. Barrett, Nigel P. Carter, Helen V. Firth, David R. Fitzpatrick, Matthew E. Hurles*, Michael Parker, Caroline F. Wright
DDD Laboratory Team
Kirsty Ambridge, Daniel M. Barrett, Tanya Bayzetinova, Susan Gribble, Netravathi Krishnappa, Laura E. Mason, Elena Prigmore, Diana Rajan
DDD Model Organisms
Eve L. Coomber, Sebastian S. Gerety
DDD Informatics Team
Stephen Clayton, Tomas W. Fitzgerald, Philip Jones, Ray Miller, Adrian R. Tivey DDD Analysis Team Nadia Akawi, Saeed Al-Turki, Jeffrey C. Barrett, Tomas W. Fitzgerald, Matthew E. Hurles, Wendy D. Jones, Daniel King, Margriet van Kogelenberg, Jeremy McRae, Katherine I. Morley, Vijaya Parthiban, Alejandro Sifrim
DDD Ethics, Social Science and Policy Team
Anna Middleton, Michael Parker, Caroline F. Wright
Wellcome Trust Sanger Institute Staff
Wellcome Trust Sanger Institute, Wellcome Trust Genome Campus, Hinxton, Cambridge, CB10 1SA, UK
DECIPHER Team:Paul Bevan, Eugene Bragin, G. Jawahar Swaminathan
WTSI Pipelines Staff (sample QC, genotyping, pulldown, sequencing, informatics)
Rob Andrews, John Burton, Suzannah J. Bumpstead, Sarah Edkins, Peter Ellis, Emma Gray, David Jones, Carol Scott, Douglas Simpkin, Danielle Walker, Sara Widaa
WTSI FISH Team
Ruby Banerjee, Beiyuan Fu, Sandra Louzada Gomes Pereira, Fentang Yang
UK NHS Regional Genetic Services (* local Principal Investigator)
Aberdeen (North of Scotland Regional Genetics Service, NHS Grampian, Department of Medical Genetics Medical School, Foresterhill, Aberdeen, AB25 2ZD, UK)
Recruiting Consultant Clinical Geneticists: John Dean*, Ruth McGowan, Alison Ross
Research Nurse/ Genetic Counsellors: Mariella D'Alessandro Diagnostic Laboratory Scientists: Paul Batstone, Shalaka Samant
Belfast (Northern Ireland Regional Genetics Centre, Belfast Health and Social Care Trust, Belfast City Hospital, Lisburn Road, Belfast, BT9 7AB, UK)
Recruiting Consultant Clinical Geneticists: Tabib Dabir, Deirdre Donnelly, Alex Magee, Vivienne McConnell, Shane McKee*, Fiona Stewart
Research Nurse/ Genetic Counsellors: Claire Kirk Diagnostic Laboratory Scientists: Mervyn Humphreys, Susan McNerlan
Birmingham (West Midlands Regional Genetics Service, Birmingham Women's NHS Foundation Trust, Birmingham Women's Hospital, Edgbaston, Birmingham, B15 2TG, UK)
Recruiting Consultant Clinical Geneticists: Louise Brueton, Trevor Cole*, Nicola Cooper, Helen Cox, Joanna Jarvis, Derek Lim, Jenny Morton, Andrew Norman, Chirag Patel, Nicola Ragge, Saba Sharif, Mark Tein, Julie Vogt, Denise Williams
Research Nurse/ Genetic Counsellors: Gail Kirby
Diagnostic Laboratory Scientists: David Bohanna, Kirsten McKay, Dominic J McMullan

Bristol (Bristol Genetics Service (Avon, Somerset, Gloucs and West Wilts), University Hospitals Bristol NHS Foundation Trust, St Michael's Hospital, St Michael's Hill, Bristol, BS2 8DT, UK)
Recruiting Consultant Clinical Geneticists: Ruth Newbury-Ecob*, Sarah Smithson Research *Nurse/ Genetic Counsellors*: Rose Hawkins
Diagnostic Laboratory Scientists: Eileen Roberts, Christopher Wragg Cambridge (East Anglian Medical Genetics Service, Box 134, Cambridge University Hospitals NHS Foundation Trust, Cambridge Biomedical Campus, Cambridge ,CB2 0QQ, UK)
Recruiting Consultant Clinical Geneticists: Ruth Armstrong, Helen Firth*, Simon Holden, Sarju Mehta, Soo-Mi Park, Joan Paterson, Lucy Raymond, Richard Sandford, Geoff Woods
Research Nurse/ Genetic Counsellors: Jonathan Roberts, Sarah Wilcox
Diagnostic Laboratory Scientists: Ingrid Simonic, Becky Treacy Cardiff (Institute Of Medical Genetics, University Hospital Of Wales, Heath Park, Cardiff, CF14 4XW, UK and Department of Clinical Genetics, Block 12, Glan Clwyd Hospital, Rhyl, Denbighshire, LL18 5UJ, UK)
Recruiting Consultant Clinical Geneticists: Hayley Archer, Sally Davies, Dhavendra Kumar, Emma McCann*, Daniela T. Pilz*, Annie Procter
Research Nurse/ Genetic Counsellors: Karenza Evans Diagnostic
Laboratory Scientists: Sian Morgan, Hood Mugalaasi Dublin (National Centre for Medical Genetics, Our Lady's Children's Hospital, Crumlin, Dublin 12, Ireland)
Recruiting Consultant Clinical Geneticists: Sally Ann Lynch*
Research Nurse/ Genetic Counsellors: Rosie O'Shea Dundee (East of Scotland Regional Genetics Service, Human Genetics Unit, Pathology Department, NHS Tayside, Ninewells Hospital, Dundee, DD1 9SY, UK)
Recruiting Consultant Clinical Geneticists: Jonathan Berg*, David Goudie, Susann Schweiger
Research Nurse/ Genetic Counsellors: Debbie Rice
Diagnostic Laboratory Scientists: David Baty, Norman Pratt Edinburgh (MRC Human Genetics Unit, MRC IGMM, University of Edinburgh, Western General Hospital, Edinburgh, EH4 2XU, UK)
Recruiting Consultant Clinical Geneticists: David R. FitzPatrick*, Wayne Lam, Anne Lampe
Research Nurse/ Genetic Counsellors: Philip Greene
Diagnostic Laboratory Scientists: Eddy Maher, David Moore
Exeter (Peninsula Clinical Genetics Service, Royal Devon and Exeter NHS Foundation Trust, Clinical Genetics Department, Royal Devon & Exeter Hospital (Heavitree), Gladstone Road, Exeter, EX1 2ED, UK)
Recruiting Consultant Clinical Geneticists: Carole Brewer, Bruce Castle, Emma Kivuva*, Julia Rankin, Charles Shaw-Smith, Claire Turner, Peter Turnpenny
Research Nurse/ Genetic Counsellors: Gemma Devlin, Sarah Everest
Diagnostic Laboratory Scientists: Sian Ellard, Carolyn Tysoe Glasgow (West of Scotland Regional Genetics Service, NHS Greater Glasgow and Clyde, Institute Of Medical Genetics, Yorkhill Hospital, Glasgow, G3 8SJ, UK)
Recruiting Consultant Clinical Geneticists: Rosemarie Davidson, Carol Gardiner, Shelagh Joss, Esther Kinning, Victoria Murday, John Tolmie*, Margo Whiteford
Research Nurse/ Genetic Counsellors: Alexis Duncan
Diagnostic Laboratory Scientists: Gordon Lowther, Nicola Williams Leeds (Yorkshire Regional Genetics Service, Leeds Teaching Hospitals NHS Trust, Department of Clinical Genetics, Chapel Allerton Hospital, Chapeltown Road, Leeds, LS7 4SA, UK)

Recruiting Consultant Clinical Geneticists: Chris Bennett, Moira Blyth*, Emma Hobson, Alison Kraus, Katrina Prescott*, Audrey Smith, Jenny Thomson
Research Nurse/ Genetic Counsellors: Miranda Squires
Diagnostic Laboratory Scientists: Andrea Coates, Sarah Hewitt, Paul Roberts
Leicester (Leicestershire Genetics Centre, University Hospitals of Leicester NHS Trust, Leicester Royal Infirmary (NHS Trust), Leicester, LE1 5WW, UK)
Recruiting Consultant Clinical Geneticists: Pradeep Vasudevan*
Research Nurse/ Genetic Counsellors: Beckie Kaemba, Sandra Kazembe
Diagnostic Laboratory Scientists: Lara Cresswell
Liverpool (Merseyside and Cheshire Genetics Service, Liverpool Women's NHS Foundation Trust, Department of Clinical Genetics, Royal Liverpool Children's Hospital Alder Hey, Eaton Road, Liverpool, L12 2AP, UK)
Recruiting Consultant Clinical Geneticists: Astrid Weber*, Alan Fryer, Lynn Greenhalgh, Elizabeth Sweeney Research Nurse/ Genetic Counsellors: Gillian Roberts, Vivienne Sutton *Diagnostic Laboratory Scientists*: Angela Douglas, Una Maye
London - North West Thames (North West Thames Regional Genetics Centre, North West London Hospitals NHS Trust, The Kennedy Galton Centre, Northwick Park And St Mark's NHS Trust Watford Road, Harrow, HA1 3UJ, UK)
Recruiting Consultant Clinical Geneticists: Birgitta Bernhard, Angela Brady, Natalie Canham*, Neeti Ghali, Susan Holder, Anthony Vandersteen, Emma Wakeling
Research Nurse/ Genetic Counsellors: Cheryl Sequeira, Roldan Singzon
Diagnostic Laboratory Scientists: Louise Bourdon, Stewart Payne
London - Great Ormond Street (North East Thames Regional Genetics Service, Great Ormond Street Hospital for Children NHS Foundation Trust, Great Ormond Street Hospital, Great Ormond Street, London, WC1N 3JH, UK)
Recruiting Consultant Clinical Geneticists: Jane Hurst*, Melissa Lees, Elisabeth Rosser, Richard Scott
Research Nurse/ Genetic Counsellors: Kate Brunstrom, Georgina Hollingsworth
Diagnostic Laboratory Scientists: Lucy Jenkins, Jonathon Waters
London – Guy's (South East Thames Regional Genetics Centre, Guy's and St Thomas' NHS Foundation Trust, Guy's Hospital, Great Maze Pond, London, SE1 9RT, UK)
Recruiting Consultant Clinical Geneticists: Fiona Connell, Charu Deshpande, Frances Flinter, Melita Irving, Dragana Josifova, Shehla Mohammed*, Leema Robert
Research Nurse/ Genetic Counsellors: Tina Fendick, Caroline Langman
Diagnostic Laboratory Scientists: Caroline Ogilvie, Michael Yau
London - St George's (South West Thames Regional Genetics Centre, St George's Healthcare NHS Trust, St George's, University of London, Cranmer Terrace, London, SW17 0RE, UK) *Recruiting Consultant Clinical Geneticists*: Frances Elmslie, Tessa Homfray, Sahar Mansour*, Meriel McEntagart, Anand Saggar, Kate Tatton-Brown
Research Nurse/ Genetic Counsellors: Uruj Anjum
Diagnostic Laboratory Scientists: Karen Marks, Rohan Taylor
Manchester (Manchester Centre for Genomic Medicine, St Mary's Hospital, Central Manchester University Hospitals NHS Foundation Trust, Manchester Academic Health Science Centre, Manchester M13 9WL)

Recruiting Consultant Clinical Geneticists: Kate Chandler, Jill Clayton-Smith*, Yanick Crow, Elizabeth Jones, Bronwyn Kerr, Kay Metcalfe
Research Nurse/ Genetic Counsellors: Carina Donnelly, Zara Skitt
Diagnostic Laboratory Scientists: Lorraine Gaunt, Emma Miles Newcastle (Northern Genetics Service, Newcastle upon Tyne Hospitals NHS Foundation Trust, Institute of Human Genetics, International Centre for Life, Central Parkway, Newcastle upon Tyne, NE1 3BZ, UK)
Recruiting Consultant Clinical Geneticists: John Burn, Richard Fisher, Judith Goodship, Alex Henderson, Tara Montgomery, Miranda Splitt*, Michael Wright
Research Nurse/ Genetic Counsellors: Linda Sneddon
Diagnostic Laboratory Scientists: David Bourn, Stephen Hellens
Nottingham (Nottingham Regional Genetics Service, City Hospital Campus, Nottingham University Hospitals NHS Trust, The Gables, Hucknall Road, Nottingham NG5 1PB, UK) *Recruiting Consultant Clinical Geneticists*: Abhijit Dixit, Jacqueline Eason*, Ajoy Sarkar, Nora Shannon, Mohnish Suri
Research Nurse/ Genetic Counsellors: Ann Selby
Diagnostic Laboratory Scientists: Gareth Cross, Katherine Martin Oxford (Oxford Regional Genetics Service, Oxford Radcliffe Hospitals NHS Trust, The Churchill Old Road, Oxford, OX3 7LJ, UK)
Recruiting Consultant Clinical Geneticists: Edward Blair, Richard Gibbons, Usha Kini*, Sue Price, Debbie Shears, Helen Stewart
Research Nurse/ Genetic Counsellors: Julie Phipps, Abigail Pridham, Hellen Purnell
Diagnostic Laboratory Scientists: Susan Clasper, Anneke Seller Sheffield (Sheffield Regional Genetics Services, Sheffield Children's NHS Trust, Western Bank, Sheffield, S10 2TH, UK)
Recruiting Consultant Clinical Geneticists: Meena Balasubramanian, Diana Johnson, Michael Parker*
Research Nurse/ Genetic Counsellors: Louise Nevitt, Stuart Ingram, Cat Taylor
Diagnostic Laboratory Scientists: Emma Shearing, Kath Smith
Southampton/Wessex (Wessex Clinical Genetics Service, University Hospital Southampton, Princess Anne Hospital, Coxford Road, Southampton, SO16 5YA, UK and Wessex Regional Genetics Laboratory, Salisbury NHS Foundation Trust, Salisbury District Hospital, Odstock Road, Salisbury, Wiltshire, SP2 8BJ, UK and Faculty of Medicine, University of Southampton)
Recruiting Consultant Clinical Geneticists: Munaza Ahmed, Diana Baralle, Amanda Collins, Nicola Foulds, Katherine Lachlan, I. Karen Temple*, Diana Wellesley
Research Nurse/ Genetic Counsellors: Lucy Harrison, Audrey Torokwa Diagnostic Laboratory scientists: David J. Bunyan, Morag N. Collinson

Molecular dynamic stimulations

To compare the structural mobility of GluA2 and its mutants we built a model of each protein ectodomain including an LBD and an ATD (Online Methods) by modelling mutations on the wild-type, and followed their behaviour along time by means of atomistic molecular dynamics simulations in water solvent. Proteins with mutations in the pore region where omitted as either the mutations were close to or included in the transmembrane domains (TMDs) which were not modelled. We aimed to ascertain the effect of the mutations on the glutamate (GLU) binding-pocket. Interestingly, in the studied mutated proteins this group of atoms appear to have a higher level of rigidity compared to the wild-type protein. While the wild-type crystal structure is symmetric, after 10 ns the conformations of pockets associated with chains C and D, which are coupled in the binding site, diverge with respect to those associated with A and B which do not change conformation. In the observed timeframe molecules underwent concerted macroscopic movements and this is reflected by minor variations in their backbone root mean squared deviation (RMSD, Supplementary Figure 9) and radius of gyration (Supplementary Figure 10).The RMSD, which is a measure of the average atoms displacement from the starting configuration, clearly indicates that amino acids in the GLU binding site are independently mobile at a timescale consistent with our simulations with two pockets reaching values larger than 0.27nm. However, the same is not true for most mutants: p.D302G, p.F644L, p.P528T and p.V647L whose RMSD do not exceed 0.22nm. The remaining mutants studied showed an intermediate behaviour.

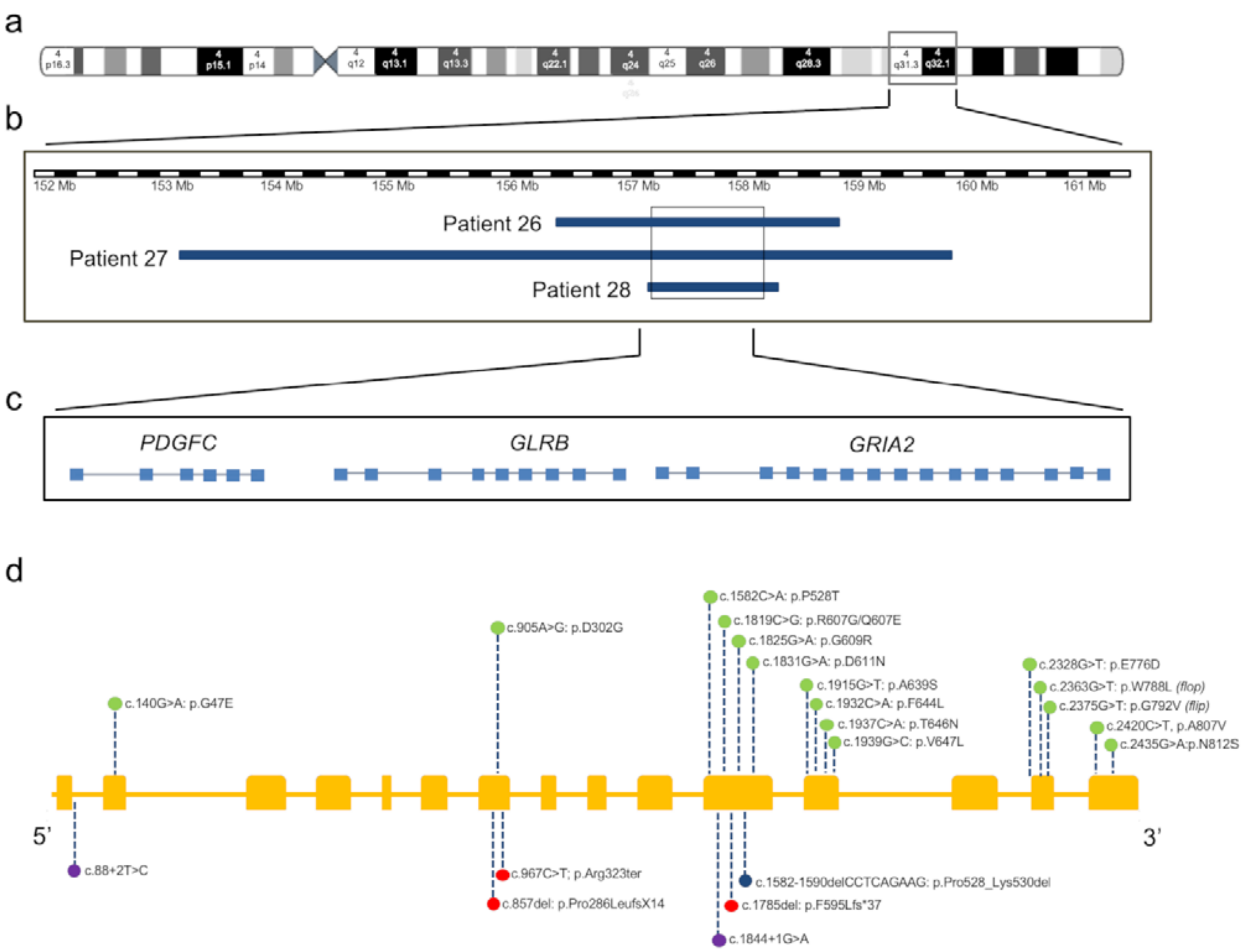

Supplementary Fig. 1. Molecular genetic findings in 28 patients with *de-novo GRIA2* variants. (**a**) Top, schematic of chromosome 4. **(b)** Middle, three microdeletions at 4q31.3 - 4q32.1 (GRCh37/hg19). **(c)** The smallest region of overlap (black rectangle) is chr4:157,343,163-158,271,008 (GRCh37/hg19) comprising three genes, *PDGFC*, *GLRB* and *GRIA2* of which GRIA2 is the one with highest pLI (Supplementary Table 2 and 3). (**d**) Schematic of the exon–intron structure of *GRIA2* (NM000826.3) indicating the positions missense variants (top, green dots), and LoF variants (below, violet dots=splice-site variants, red= frameshift variants, blue = in-frame deletion).

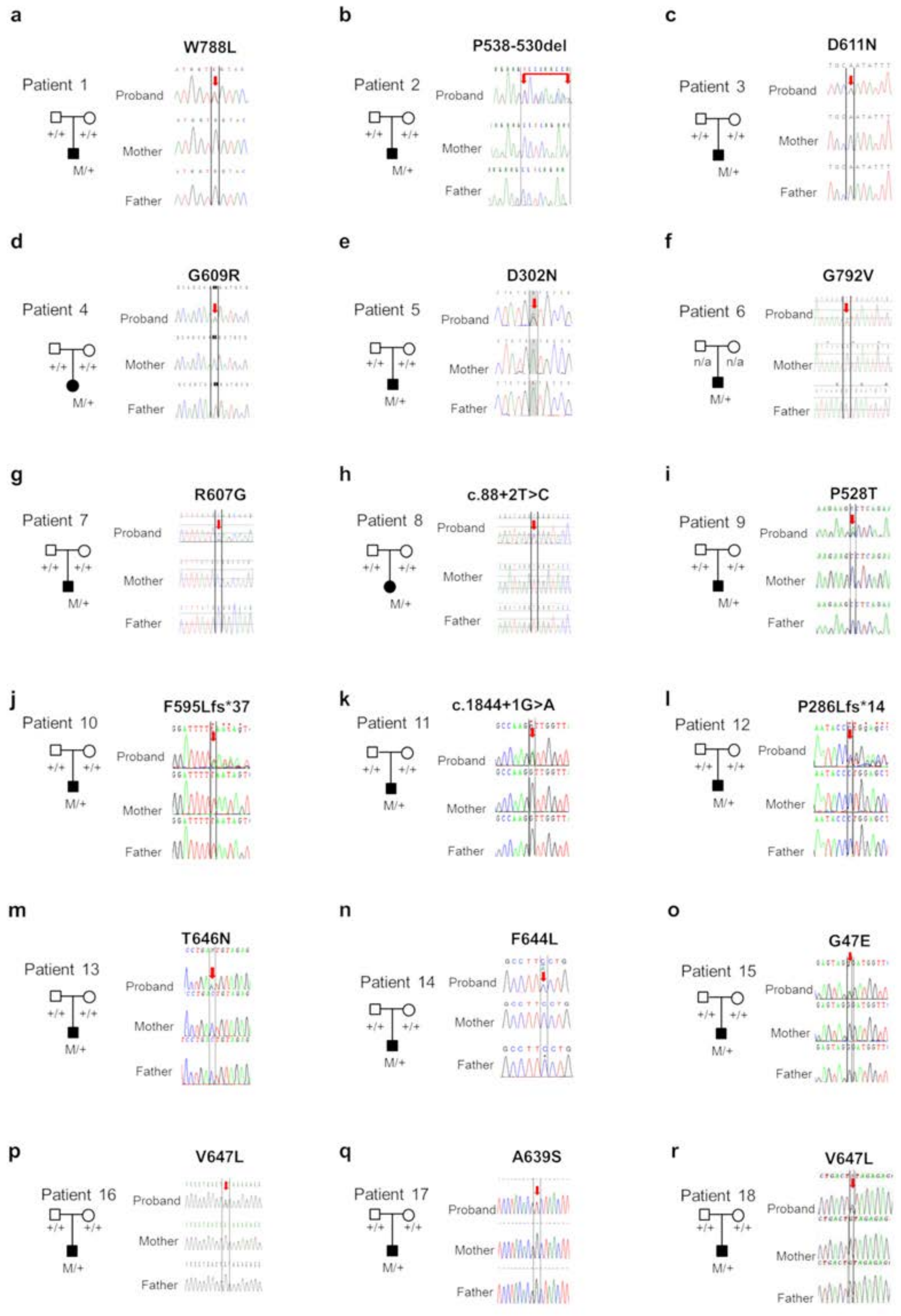

Supplementary Figure2. Sanger sequences of kindreds with *de-novo GRIA2* intragenic variants.

Chromatograms of patients 1-18 (a-r) and their parents confirm the *de-novo* occurrence of the *GRIA2* variants in all cases. M/+ denotes the indicated novel *GRIA2* variant in the heterozygous state, and +/+ denotes homozygous wild-type sequence. Mutant bases are indicated by a red arrow.

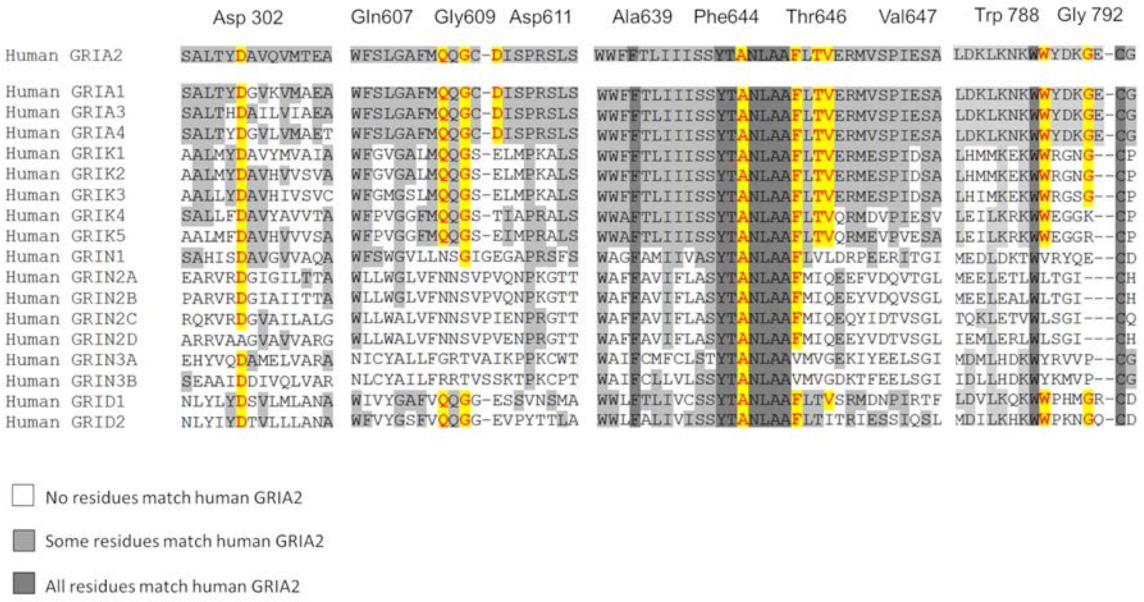

Supplementary Figure 3. Multiple alignments of the amino acids altered by the identified *GRIA2* intragenic variants across glutamate receptor subunit homologue genes.

Multiple alignments showing partial to complete conservation the mutated GluA2 residues (bold in red and highlighted in yellow) across homologue ionotropic glutamate receptor subunits. All the intragenic missense variants identified in this study affect conserved residues of GluA2 ($GERP^{++}$ > 4, see Supplementary Table 1). Human GRIA2 (NP_000817.2), human GRIA1 (NP_000818.2), GRIA3 (NP_015564.4), GRIA4 (NP_000820.3), GRIN1 (NP_015566.1), GRIN2A (NP_001127879.1), GRIN2B (NP_000825.2), GRIN2C (NP_000826.2), GRIN2D (NP_000827.2), GRIN3A (NP_597702.2), GRIN3B (NP_619635.1). For the W788L variant alignment we used $GluA2^{flop}$ isoform (NP_001077088.1)

a

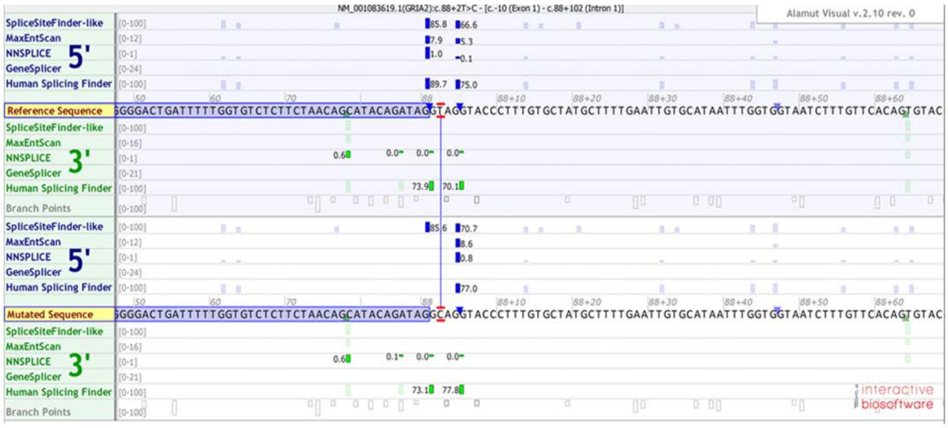

b

	SSF [0-100]	MaxEnt [0-12]	NNSPLICE [0-1]	GeneSplicer [0-24]	HSF [0-100]
Threshold	*≥ 0*	*≥ 0*	*≥ 0*	*≥ 0*	*≥ 0*
Exon 1 – c.2	= 65.90				
Exon 1 – c.14	= 59.57				
Exon 1 – c.24	= 35.50		= 0.00		= 54.08
Exon 1 – c.39	= 40.70		= 0.00		= 55.90
Exon 1 – c.61	= 60.13		= 0.00		= 71.29
Exon 1 – c.63	= 42.62		= 0.00		= 56.67
Exon 1 – c.76	= 44.51				
**Exon 1 – c.88 N**	**85.79 ⇒ 85.58 (-0.2%)**	**7.92 ⇒ —**	**0.99 ⇒ —**		**89.68 ⇒ —**
Intron 1 – c.88+4	**66.61 ⇒ 70.74 (+6.2%)**	**5.35 ⇒ 8.63 (+61.5%)**	**0.11 ⇒ 0.82 (+641.9%)**		**75.03 ⇒ 77.01 (+2.6%)**
Intron 1 – c.88+13	= 41.88		= 0.00		= 57.78
Intron 1 – c.88+15	= 38.16				
Intron 1 – c.88+20	= 47.37				
Intron 1 – c.88+31	= 51.27		= 0.00		= 61.30
Intron 1 – c.88+33	= 39.85				
Intron 1 – c.88+43	= 55.20		= 0.00		= 69.12
Intron 1 – c.88+46	= 70.26	= 3.00	= 0.06		= 79.00
Intron 1 – c.88+55	= 36.15		= 0.00		= 54.23
Intron 1 – c.88+62	= 46.18		= 0.00		= 59.41
Intron 1 – c.88+64	= 49.85		= 0.01		= 60.02
Intron 1 – c.88+80	= 60.09		= 0.00		= 69.86
Intron 1 – c.88+85	= 59.37		= 0.00		= 66.45
Intron 1 – c.88+95	= 55.65				= 67.47

Supplementary Figure 4. Predicted effect of splice-site c.88+2T>C *de-novo GRIA2* variant

(a) Prediction programs Splice Site Finder-like, MaxEntScan, NNSplice, Gene Splicer, and Human Splicing Finder compiled by Alamut Visual v2.9 rev. 0 (Interactive Bio software, Rouen, France) indicate that the splicing variant c.88+2T>C in *GRIA2* results in an abolishment of the natural donor side in exon 1 and strengthening of an alternative donor splice site at position c.88+4. (b) In silico analysis of *GRIA2* c.88+2T>C effect on donor sites [c.88+102 (Intron 1)].

a

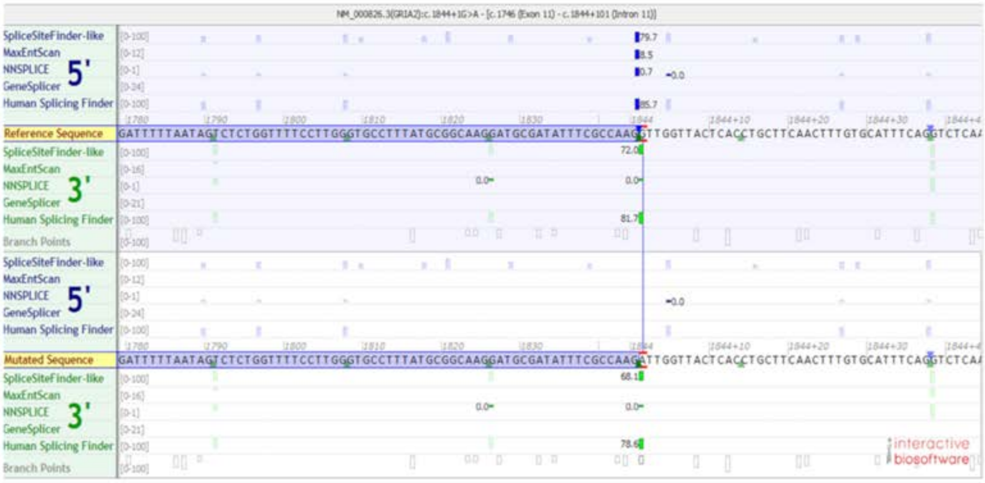

b

	SSF [0-100]	MaxEnt [0-12]	NNSPLICE [0-1]	GeneSplicer [0-24]	HSF [0-100]
Threshold	*≥ 0*	*≥ 0*	*≥ 0*	*≥ 0*	*≥ 0*
Exon 11 – c.1753	= 60.07		= 0.01		= 65.88
Exon 11 – c.1756	= 59.78		= 0.00		= 69.59
Exon 11 – c.1789	= 46.29		= 0.00		= 57.88
Exon 11 – c.1796	= 53.27		= 0.00		= 66.93
Exon 11 – c.1807	= 59.07		= 0.00		= 71.19
Exon 11 – c.1809	= 37.99				
Exon 11 – c.1817	= 40.11				
Exon 11 – c.1820	= 78.07				
Exon 11 – c.1828	= 51.94				
Exon 11 – c.1838	= 38.68				
**Exon 11 – c.1844 N**	**79.74 ⇒ —**	**8.46 ⇒ —**	**0.66 ⇒ —**		**85.68 ⇒ —**
Intron 11 – c.1844+4	= 64.79		**0.00 ⇒ 0.01 *(+347.2%)***		= 76.23
Intron 11 – c.1844+15	= 32.27				
Intron 11 – c.1844+26	= 47.45		= 0.00		= 60.27
Intron 11 – c.1844+28	= 47.95				
Intron 11 – c.1844+37	= 61.92		= 0.01		= 71.82
Intron 11 – c.1844+44	= 55.49		= 0.00		= 65.58
Intron 11 – c.1844+57	= 64.25		= 0.02		= 72.01
Intron 11 – c.1844+59	= 43.17		= 0.00		= 56.52
Intron 11 – c.1844+82	= 56.46		= 0.00		= 68.45
Intron 11 – c.1844+89	= 32.89				
Intron 11 – c.1844+92	= 30.20				

Supplementary Figure 5. Predicted effect of splice-site c.1844+1G>A *de-novo GRIA2* variant
(a) Prediction programs Splice Site Finder-like, MaxEntScan, NNSplice, Gene Splicer, and Human Splicing Finder compiled by Alamut Visual v2.9 rev. 0 (Interactive Bio software, Rouen, France) indicate that the splicing variant c.1844+1G>A in *GRIA2* results in a complete loss of donor splice site of exon 11. (b) in silico analysis of *GRIA2* c.1844+1G>A effect on donor sites [c.1746 (Exon 11) c.1844+101 (Intron 11)].

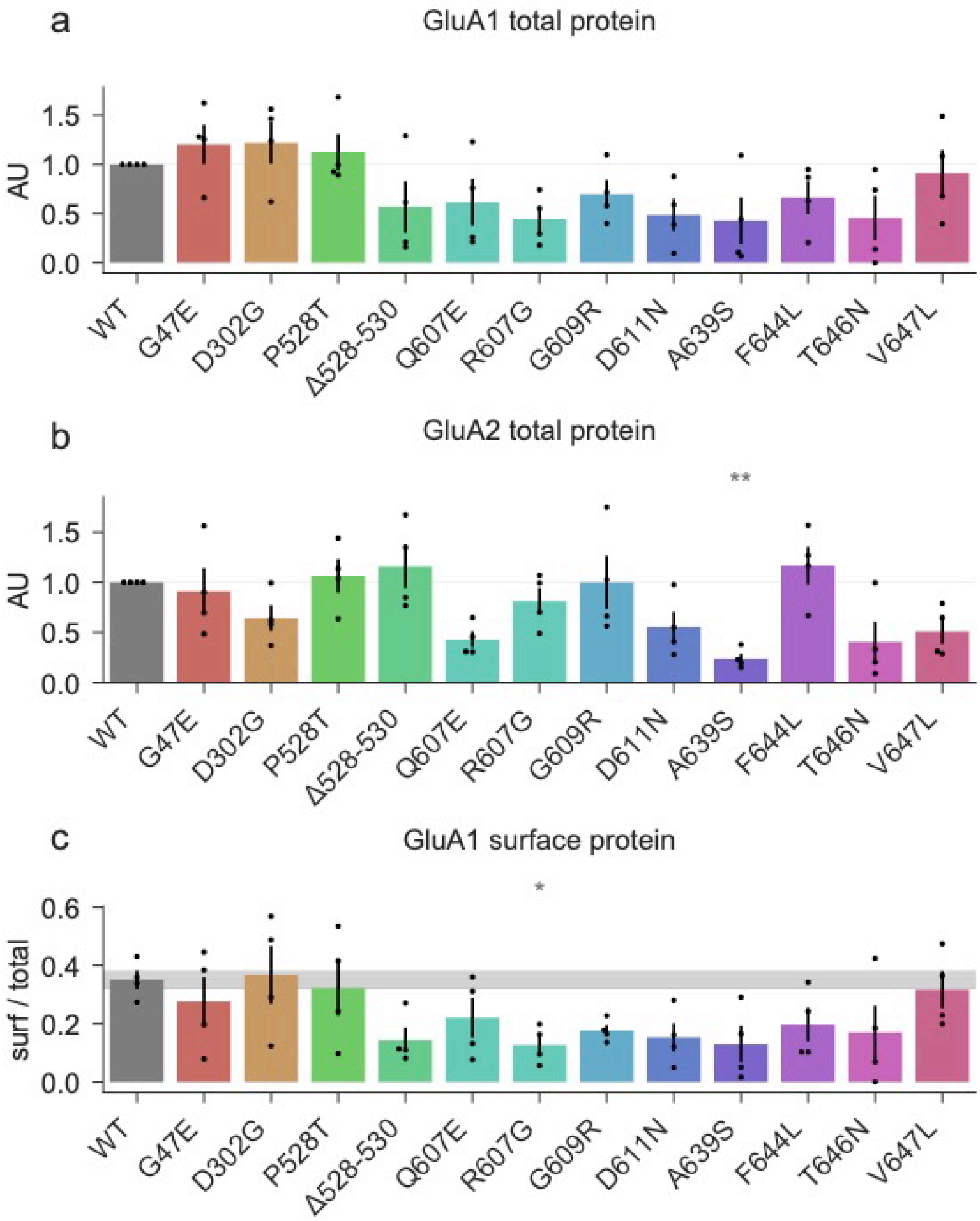

Supplementary Figure 6. Additional quantification of western blots.
Total cell input and biotinylated pull-down fraction were quantified relative to GADPH and then normalised to a WT control on the same gel for both GluA1 **(a)** and GluA2 **(b)**. Surface protein was quantified as PD/IN for GluA1 **(c)** * $p<0.05$, ** $p<0.01$ vs WT.

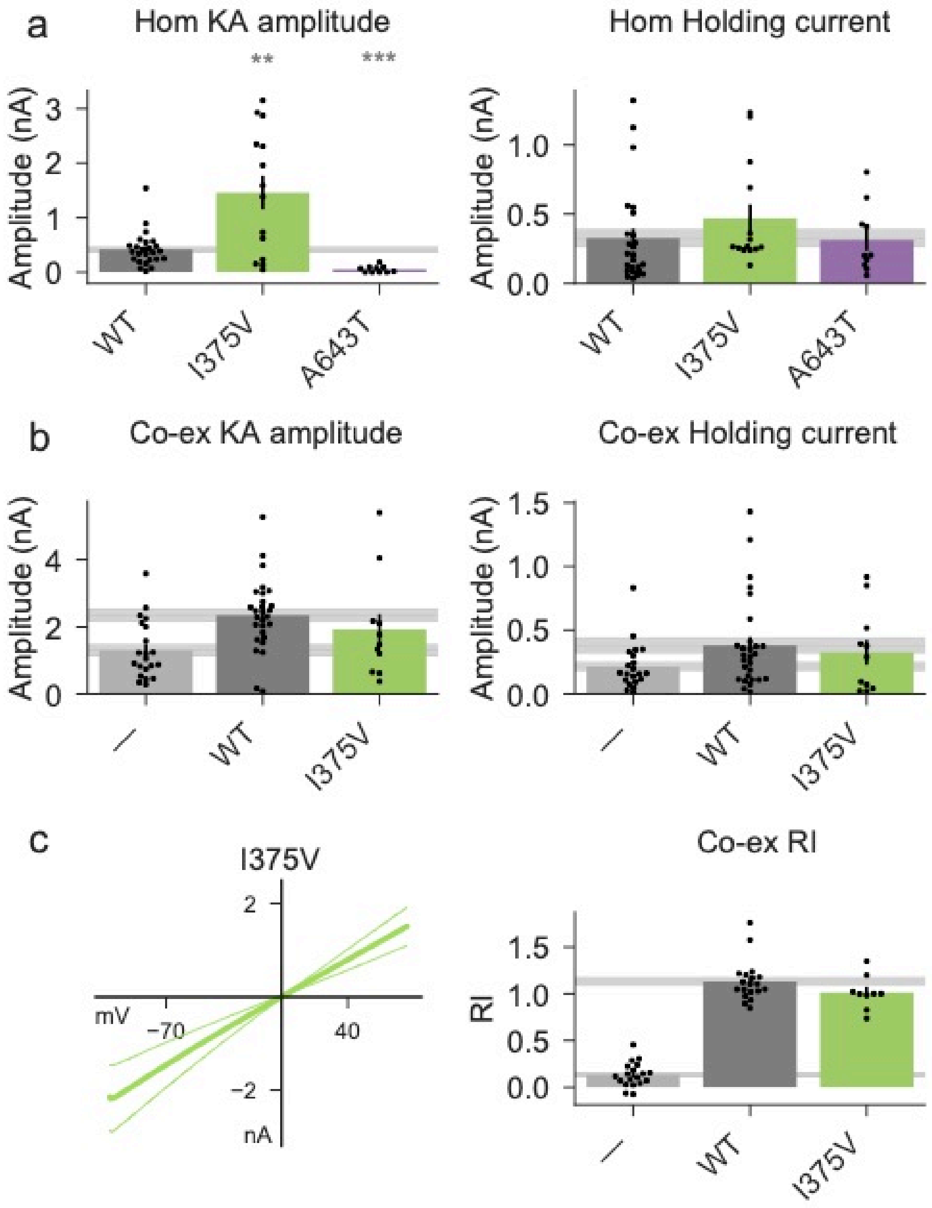

Supplementary Figure 7. Additional non-clinical mutants included for comparison.
We tested I375V, the most common variant of GluA2 not associated with a specific disease, and A643T, which is homologous to the Lurcher mutation which has been studied in other iGluRs. **(a)** Homomeric GluA2 had altered maximal current for both mutations. Holding current was not affected. Co-expression of I375V with GluA1 did not reveal differences in amplitude **(b)** or rectification.

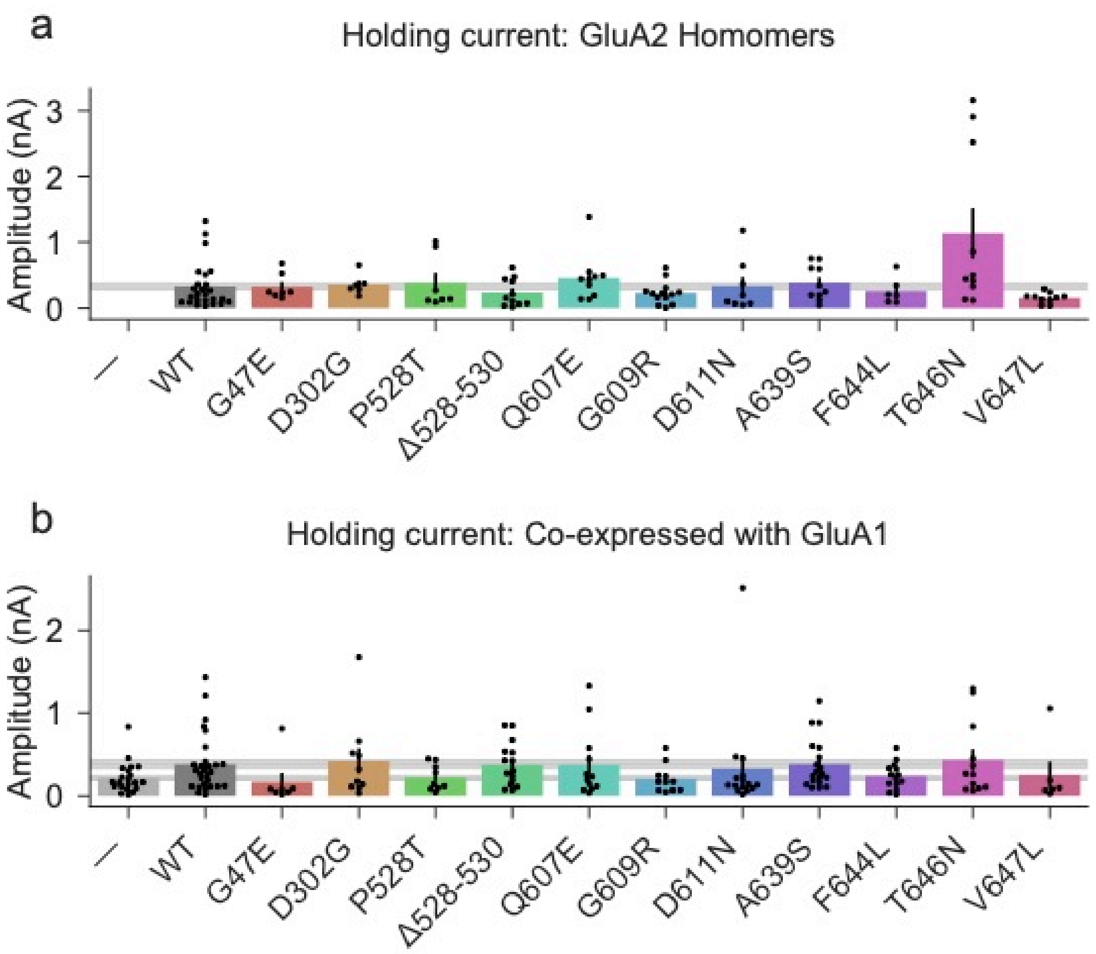

Supplementary Figure 8. Holding currents do not suggest large leak currents generated by mutant GluA2 subunits. Holding current for HEK cell expressing GluA2 and stargazin **(a)**, or GluA1, GluA2 and stargazin **(b)**, voltage clamped at -74 mV. No significant differences detected compared to positive or negative controls.

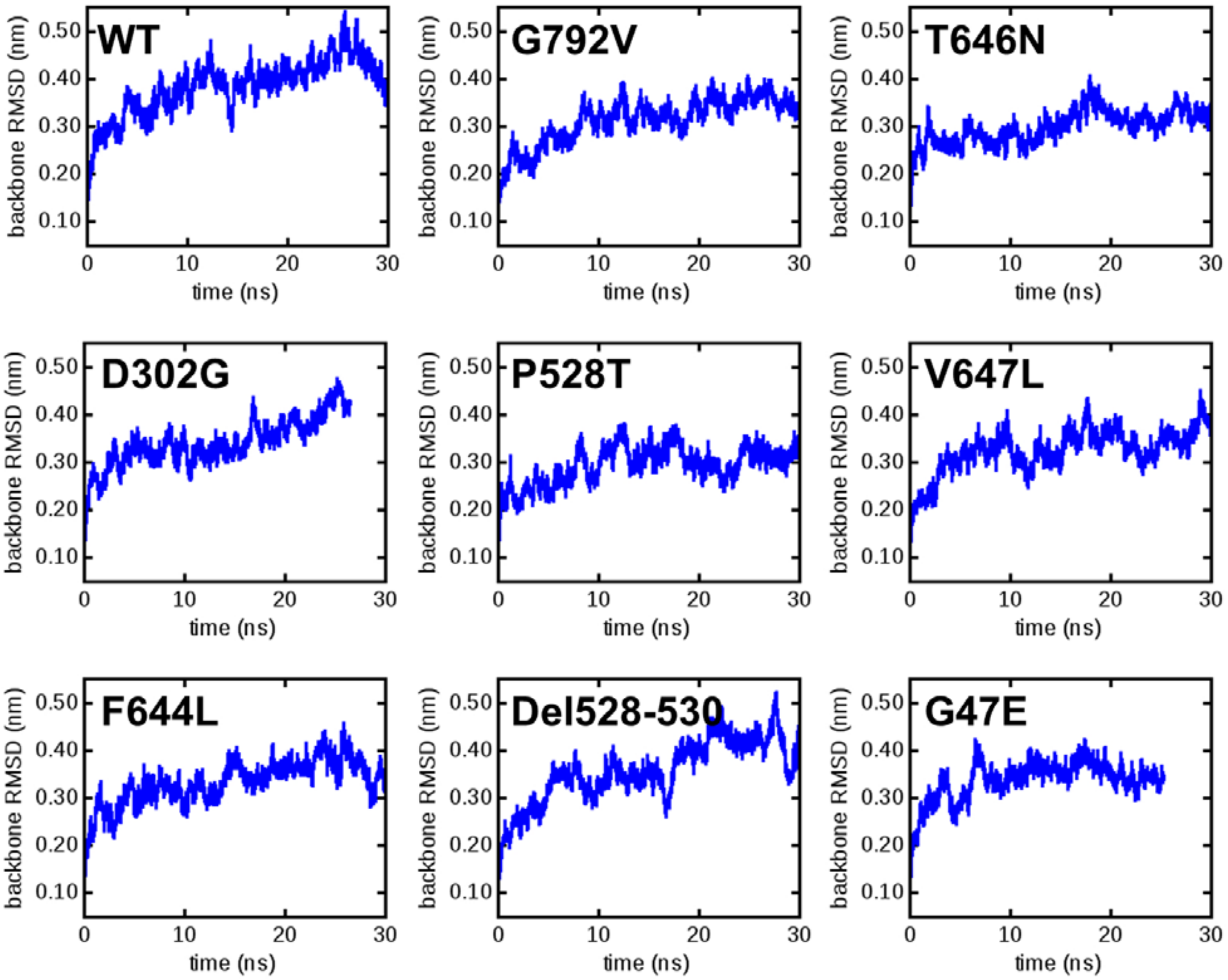

Supplementary Figure 9. Comparison of Root Mean Square Deviation (RMSD) of the Glutamate binding pocket in GluA2 WT and 8 intragenic mutants. Root Mean Square Deviation (RMSD) of the GluA2 Glutamate binding pocket (GLU) amino acids along the simulated time for the wild-type protein and *GRIA2* mutants. Dynamic stimulations show RMSD of the GluA2 GLU binding amino acids along the simulated time for the wild type protein and the mutants p.G47E, p.D302G, p.P528T, p.P528_K530del, p. F644L, p.T646N, p.V647L, p.G792V

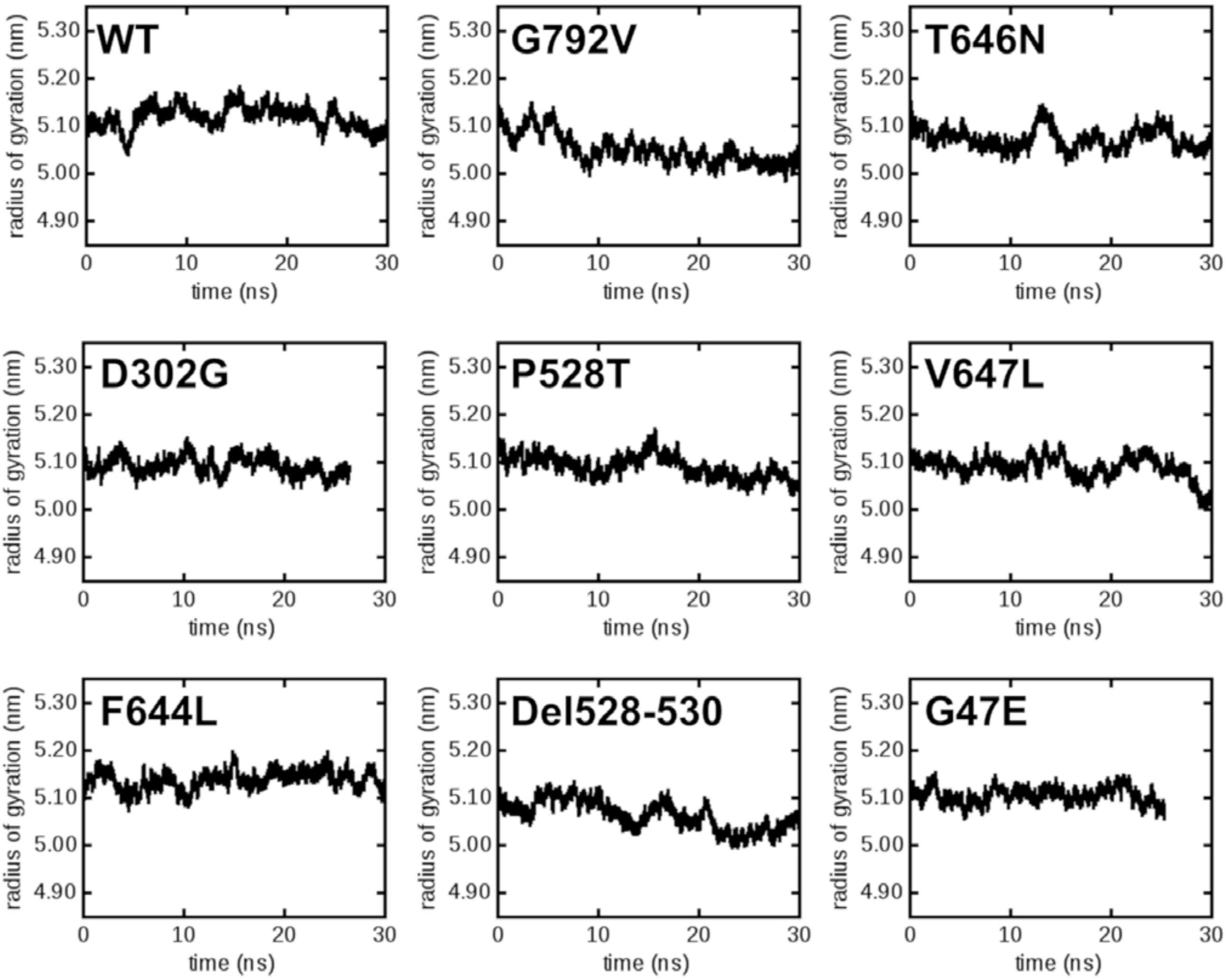

Supplementary Figure 10. Comparison of Radius of gyration along the simulated time for GluA2 wild-type and 8 intragenic mutants. Dynamic stimulations show Radius of gyration along simulated time for the wild-type GluA2 protein and the mutants p.G47E, p.D302G, p.P528T, p.P528_K530del, p. F644L, p.T646N, p.V647L, p.G792V

Patient	Age/Gender	Variant	OFC	DD	ID	Main seizure types	ASD	RTT-like features	Speech	Brain imaging	Other features
1	3.5 years; M	p.Trp788Leu	<3rd	+++	+++	Focal, tonic-clonic	n/a	Head growth deceleration	Absent	Cerebellar atrophy	Unable to walk
2	13 years; F	p.Pro528_Lys530del	25th	++	+++	No	Yes	Stereotyped hand movements	Delayed	Normal	n/a
3	19 years; M	p.Asp611Asn	25th	++	+++	No	Yes	Language regression	Absent	Normal	Obsessive-compulsive traits
4	19 years; F	p.Gly609Arg	25th -50th	++	+++	No	No	Psychomotor regression	Absent	White matter changes	Ataxic gait, dystonia
5	10 years; M	p.Asp302Gly	50th	+	++	Febrile	Yes	n/a	Delayed	Normal	n/a
6	31 years; F	p.Gly792Val	<3rd	++	++	No	Yes	Head growth deceleration	Delayed	n/a	Self-harm behaviour
7	11 years; F	p.Arg607Gly	n/a	++	+++	Tonic-clonic	Yes	Stereotyped hand movements	Absent	n/a	Unable to walk
8	2 years; M	c.88+2T>C; p.?	75th- 90th	++	++	No	n/a	No	n/a	Normal	Cryptorchidism
9	9 years; M	p.Pro528Thr	10th	++	++	No	Yes	Stereotyped hand movements	Delayed	Normal	Joint hyper-extensibility
10	5 years; F	p.Phe595LeufsX37	10th- 25th	++	++	No	Yes	n/a	Delayed	Normal	n/a
11	8 years; M	c.1844+1G>A; p.?	5-10th	+	++	No	Yes	Microcephaly	Delayed	Normal	Hyperactive behaviour
12	6 years; M	p.Pro286LeufsX14	25th-50th	++	+++	No	Yes	n/a	Absent	Normal	Obsessive-compulsive traits
13	3 years; F	p.Thr646Asn	<3rd	++	++	Focal, clonic	No	Head growth deceleration	Absent	Cerebellar atrophy	Dystonia, dyskinesia
14	8 years; F	p.Phe644Leu	n/a	++	++	No	Yes	No purposeful hand movements	Absent	Normal	Sleep disturbances
15	13 years; F	p.Gly47Glu	n/a	++	++	No	Yes	No	Delayed	n/a	n/a
16	9 years; M	p.Val647Leu	25th	++	+++	Focal, tonic-clonic	n/a	Psychomotor regression	Absent	Mild cerebral atrophy	Unable to walk
17	3 months†; M	p.Ala639Ser	10th	++	n/a	Focal, tonic-clonic	n/a	Head growth deceleration	n/a	Cerebellar atrophy	Exaggerated startle response
18	5 years; M	p.Val647Leu	10th	+++	++	Tonic-clonic	Yes	No	Absent	Brain/cerebellar atrophy	Spasticity, chorea
19	5 years; M	p.Arg323ter	n/a	++	++	No	Yes	Hand and finger mannerisms	Delayed	n/a	Obsessive- compulsive traits
20	5 months†; F	p.Ala639Ser	50th-75th	++	n/a	Focal, clonic	n/a	n/a	n/a	Brain/cerebellar atrophy	Breathing difficulties
21	3 years; M	p.Val647Leu	>3rd	++	++	Focal, tonic-clonic	n/a	Head growth deceleration	Absent	Normal	n/a
22	3 years; M	p.Asn812Ser	90th	++	++	No	Yes	Hand clapping	Delayed	Normal	Repetitive behaviours
23	30 years; F	p.Glu776Asp	n/a	++	++	Tonic	Yes	No	Absent	Normal	Repetitive behaviors
24	5 years ; M	p.Val647Leu	n/a	+++	+++	Focal, clonic	n/a	No	Absent	Brain/cerebellar atrophy	Unable to walk
25	3.6 years; F	p.Ala807Val	50th	++	++	Tonic-clonic, focal	Yes	Stereotyped hand movements	Absent	Normal	n/a
26	6 years; F	4q32.1del (156,351,739-158,983,901)x1dn	10th –25th	++	++	No	Yes	No	Delayed	No	Joint hyper-laxity
27	12 years; F	4q32.1del (156,351,739-158,983,901)x1dn	n/a	++	++	No	No	No	Absent	n/a	Congenital clubfoot
28	13 years; M	4q32.1del (156,351,739-158,271,008)x1dn	25th	+	++	No	No	n/a	Delayed	No	Impaired social interaction

Supplementary Table 1. Phenotype of 28 individuals carrying *de-novo GRIA2* variants
OFC=Occipito-frontal circumference; DD=Developmental delay; ID=Intellectual disability; ASD=Autism spectrum disorder; RTT=Rett syndrome; †deceased

Patient	Genomic coordinates (GRCh37/hg19)	Transcript	Variant	dbSNP138	1000G	ExAC_ ALL	Gnom AD	SIFT	PolyPhen	Mutation Taster	CADD_ PHRED	GERP++
1	4:158282233-158282233	NM001083619.1	c.2363G>T; p.Trp788Leu	-	-	-	-	D(0)	D(0.999)	D(1)	15.07	5.82
2	4:158257636-158257645	NM000826.3	c.1582_1590del; p.Pro528_Lys530del	-	-	-	-	-	-	-	-	-
3	4:158257886-158257886	NM000826.3	c.1831G>A; p.Asp611Asn	-	-	-	-	D(0.01)	D(0.999)	D(1)	29.8	4.82
4	4:158257880-158257880	NM000826.3	c.1825G>A; p.Gly609Arg	-	-	-	-	D(0)	D(1)	D(1)	29.7	5.66
5	4:158253993-158253993	NM000826.3	c.905A>G; p.Asp302Gly	-	-	-	-	D(0)	D(0.78)	D(1)	28.5	5.22
6	4:158282773-158282773	NM000826.3	c.2375G>T; p.Gly792Val	-	-	-	-	D(0)	D(1)	D(1)	26.3	5.61
7	4:158257874-158257874	NM000826.3	c.1819C>G; p.Arg607Gly	-	-	-	-	D(0)	B(0.054)	D(0.99)	19.71	4.82
8	4:158142284-158142284	NM000826.3	c.88+2T>C; p.?	-	-	-	-	-	-	-	-	-
9	4:158257637-158257637	NM000826.3	c.1582C>A; p.Pro528Thr	-	-	-	-	D(0)	D(0.999)	D(1)	27.1	5.46
10	4:158257839-158257840	NM000826.3	c.1785del; p.Phe595LeufsX37	-	-	-	-	-	-	-	-	-
11	4:158257900-158257900	NM000826.3	c.1844+1G>A; p.?	-	-	-	-	-	-	-	25.7	-
12	4:158242725-158242726	NM000826.3	c.857del; p.Pro286LeufsX14	-	-	-	-	-	-	-	-	-
13	4:158262508-158262508	NM000826.3	c.1937C>A; p.Thr646Asn	-	-	-	-	D(0)	D(0.998)	D(1)	27.7	5.53
14	4:158262503-158262503	NM000826.3	c.1932C>A; p.Phe644Leu	-	-	-	-	D(0)	D(0.99)	D(1)	32	5.53
15	4:158142870-158142870	NM000826.3	c.140G>A; p.Gly47Glu	-	-	-	-	B(0.14)	D(0.942)	D(1)	24.1	5.5
16	4:158262510-158262510	NM000826.3	c.1939G>C; p.Val647Leu	-	-	-	-	D(0)	D(0.994)	D(1)	27.6	5.53
17	4:158262486-158262486	NM000826.3	c.1915G>T; p.Ala639Ser	-	-	-	-	D(0.01)	D(0.997)	D(1)	27.9	5.53
18	4:158262510-158262510	NM000826.3	c.1939G>C; p.Val647Leu	-	-	-	-	D(0)	D(0.994)	D(1)	27.6	5.53
19	4:158254055-158254055	NM000826.3	c.967C>T; p.Arg323ter	rs143505003	-	-	-	-	-	-	37	-
20	4:158262486-158262486	NM000826.3	c.1915G>T; p.Ala639Ser	-	-	-	-	D(0.01)	D(0.997)	D(1)	27.9	5.53
21	4:158262510-158262510	NM000826.3	c.1939G>C; p.Val647Leu	-	-	-	-	D(0)	D(0.994)	D(1)	27.6	5.53
22	4:158283979-158283979	NM000826.3	c.2435A>G; p.N812S	-	-	-	-	D(0)	D(0.994)	D(1)	23.9	6.08
23	4:158282726-158282726	NM000826.3	2328G>T; p.Glu776Asp	-	-	-	-	(D)0.02	(D)0.994	D(1)	25.7	6.07
24	4:158262510-158262510	NM000826.3	c.1939G>C; p.Val647Leu	-	-	-	-	D(0)	D(0.994)	D(1)	27.6	5.53
25	4:157362812-157362812	NM000826.3	c.2420C>T, p.A807V	-	-	-	-	D(0.01)	D(0.886)	D(1)	25.3	6.08

Supplementary Table 2. *GRIA2* intragenic variants identified in our cohort. B: tolerated (in SIFT), benign (in PolyPhen), D: damaging (in SIFT), probably damaging (in PolyPhen), disease causing Mutation Taster)

Gene	Genomic Position (GRCh37/hg19)	Description	Disease- associated (OMIM)	NDD- associated (DDG2P)	Haploinsufficiency Score (HI index)	Loss Intolerance (pLI – ExAC)
GRIA2	4:158125334-158287227	glutamate ionotropic receptor AMPA type subunit 2	-	-	4.81	1
PDGFC	4:157681606-157892546	platelet derived growth factor C	-	-	27.48	0.97
GLRB	4:157997209-158093242	glycine receptor beta		-	35.64	0.03
GUCY1A3	4:156587863-156653501	guanylate cyclase 1 soluble subunit alpha 1		-	44.49	0
GUCY1B3	4:156680144-156728743	guanylate cyclase 1 soluble subunit beta 1	-	-	33.12	0
ASIC5	4:156750881-156787425	acid sensing ion channel subunit family member 5	-	-	68.79	0
CTSO	4:156845270-156875069	cathepsin O	-	-	71.36	0
TDO2	4:156775890-156841558	tryptophan 2,3-dioxygenase		-	58.09	0

Supplementary Table 3. Genes in chr4: 156,351,739-158,983,901 deleted in Patient 25 (Decipher ID: 328135)

Genes in chr4:156,351,739-158,983,901 ordered from the highest to the lowest Loss Intolerance (pLI – ExAC) scores. Genes most intolerant to loss-of-function are highlighted in the blue rectangle.

Gene	Genomic Position (GRCh37/hg19)	Description	Disease- associated (OMIM)	NDD- associated (DDG2P)	Haploinsufficiency Score (HI index)	Loss Intolerance (pLI – ExAC)
FBXW7	4:153242410-153457253	F-box and WD repeat domain containing 7	-	-	2.05	1
FNIP2	4:159690290-159829201	folliculin interacting protein 2	-	-	64	1
GRIA2	4:158125334-158287227	glutamate ionotropic receptor AMPA type subunit 2	-	-	4.81	1
KIAA0922	4:154387498-154557863	transmembrane 131 like	-	-	61.39	1
PLRG1	4:155456158-155471587	pleiotropic regulator 1	-	-	9.04	1
PDGFC	4:157681606-157892546	platelet derived growth factor C	-	-	27.48	0.97
TRIM2	4:154073494-154260472	tripartite motif containing 2		-	21.8	0.89
C4orf46	4:159587831-159593407	chromosome 4 open reading frame 46	-	-	77.84	0.26
FGB	4:155484108-155492238	fibrinogen beta chain	Yes	-	48.24	0.2
NPY2R	4:156129781-156138230	neuropeptide Y receptor Y2	-	-	37.43	0.19
LRAT	4:155548097-155674270	lecithin retinol acyltransferase		Probable	31.38	0.1
FGG	4:155525286-155534119	fibrinogen gamma chain	Yes	-	52.44	0.08
SFRP2	4:154701744-154710272	secreted frizzled related protein 2	-	-	2.09	0.05
GLRB	4:157997209-158093242	glycine receptor beta		-	35.64	0.03
RBM46	4:155702365-155749965	RNA binding motif protein 46	-	-	13.21	0.02
FHDC1	4:153857504-153900848	FH2 domain containing 1	-	-	77.87	0.02
RNF175	4:154631277-154681387	ring finger protein 175	-	-	70.32	0
TLR2	4:154622652-154626851	toll like receptor 2	Yes	-	44.43	0
TMEM154	4:153539784-153601317	transmembrane protein 154	-	-	95.25	0
MAP9	4:156263810-156298122	microtubule associated protein 9	-	-	69.74	0
GUCY1A3	4:156587863-156653501	guanylate cyclase 1 soluble subunit alpha 1		-	44.49	0
GUCY1B3	4:156680144-156728743	guanylate cyclase 1 soluble subunit beta 1	-	-	33.12	0

ASIC5	4:156750881-156787425	acid sensing ion channel subunit family member 5	-	-	68.79	0
DCHS2	4:155153399-155412930	dachsous cadherin-related 2	-	-	70.72	0
CTSO	4:156845270-156875069	cathepsin O	-	-	71.36	0
MND1	4:154265801-154336270	meiotic nuclear divisions 1	-	-	6.27	0
FGA	4:155504278-155511918	fibrinogen alpha chain	Yes	-	76.19	0
C4orf45	4:159814286-159959912	chromosome 4 open reading frame 45	-	-	87.78	0
FAM198B	4:159045626-159094470	family with sequence similarity 198 member B	-	-	41.26	0
TMEM144	4:159122756-159176563	transmembrane protein 144	-	-	56.91	0
RXFP1	4:159236463-159574524	relaxin family peptide receptor 1	-	-	37.66	0
ARFIP1	4:153701089-153839615	ADP ribosylation factor interacting protein 1	-	-	13.23	0
ETFDH	4:159593277-159630775	electron transfer flavoprotein dehydrogenase		Y	46.49	0
PPID	4:159630286-159644548	peptidylprolyl isomerase D	-	-	42.35	0
TIGD4	4:153690506-153700916	tigger transposable element derived 4	-	-	42.37	0
TDO2	4:156775890-156841558	tryptophan 2,3-dioxygenase		-	58.09	0

Supplementary Table 4. Genes in chr4:153,038,154-159,949,244 deleted in Patient 26 (Decipher ID: 269176)
Genes in chr4: 153,038,154-159,949,244 ordered from the highest to the lowest **Loss Intolerance (pLI – ExAC)** scores. Genes most intolerant to loss-of-function are highlighted in the blue rectangle

Gene	Genomic Position (GRCh37/hg19)	Description	Disease- associated (OMIM)	NDD- associated (DDG2P)	Haploinsufficiency Score (HI index)	Loss Intolerance (pLI – ExAC)
GRIA2	4:158125334-158287227	glutamate ionotropic receptor AMPA type subunit 2	-	-	4.81	1
PDGFC	4:157681606-157892546	platelet derived growth factor C	-	-	27.48	0.97
GLRB	4:157997209-158093242	glycine receptor beta		-	35.64	0.03

Supplementary Table 5. Genes in chr4:157,343,163-158,271,008 deleted in Patient 27 (Decipher ID: 296516)

Genes in chr4: 157,343,163-158,271,008 ordered from the highest to the lowest Loss Intolerance (pLI – ExAC) scores. Genes most intolerant to loss-of-function are highlighted in the blue rectangle Chr4: 157,343,163-158,271,008 also corresponds to the smallest deleted region overlapping Patients 20-22 microdeletions

Clinical features			Patient 2 p.P528_K530del	Patient 3 p.D611N	Patient 4 p.G609R	Patient 6 p.G792V	Patient 7 p.R607G	Patient 14 p.F644L
Major RTT criteria		Partial or complete loss of acquired purposeful hand skills	-	-	-	-	-	-
Major RTT criteria		Partial or complete loss of acquired spoken language	-	+	-	-	-	-
Major RTT criteria		Gait abnormalities including gait dyspraxia and inability to walk	-	-	+	+	+	+
Major RTT criteria		Stereotypic hand movements including hand wringing, hand clapping, mouthing and rubbing automatisms	+	+	+	-	+	hands to mouth
Supportive RTT criteria	Growth	Growth retardation		n/a		+	-	-
Supportive RTT criteria	Growth	Microcephaly (or deceleration of head growth)	+	-	-	+	n/a	n/a
Supportive RTT criteria	Seizures/sleep	Seizures	-	-	n/a	-	+	-
Supportive RTT criteria	Seizures/sleep	Impaired sleep pattern	+	-	+	-	-	+
Supportive RTT criteria	Behavioral	Stereotyped hand movements	+	+	+	-	+	hands to mouth
Supportive RTT criteria	Behavioral	Autistic features	+	+	-	+	+	+
Supportive RTT criteria	Behavioral	Bruxism when awake	-	-	+	n/a	-	-
Supportive RTT criteria	Behavioral	Inappropriate laughing/screaming spells	+	+	-	+	-	+
Supportive RTT criteria	Behavioral	Breathing disturbances when awake	-	+	-	-	+	-
Supportive RTT criteria	Motor/sensory	Abnormal muscle tone	-	-	+	-	+	+
Supportive RTT criteria	Motor/sensory	Diminished response to pain	n/a	-	n/a	n/a	n/a	n/a
Supportive RTT criteria	Motor/sensory	Gait dyspraxia or absent gait	-	-	+	+	+	+
Supportive RTT criteria	Autonomic	Peripheral vasomotor disturbances	-	-	-	-	-	-
Supportive RTT criteria	Autonomic	Small cold hands/feet	-	-	-	n/a	-	-
Supportive RTT criteria	Gastrointestinal	Constipation	-	-	-	-	-	-
Supportive RTT criteria	Skeletal	Scoliosis/kyphosis	n/a	-	-	-	-	-

Supplementary Table 6. RTT-like features observed in GRIA2 NDD

	Patient 1 (p.Trp788Leu)	Patient 7 (p.Arg607Gly)	Patient 13 (p.Thr646Asn)	Patient 16 (p.Val647Leu)	Patient 17 (p.Ala639Ser)	Patient 18 (p.Val647Leu)	Patient 20 (p.Ala639Ser)	Patient 21 (p.Val647Leu)	Patient 24 (p.Val647Leu)
Seizure onset	Neonatal	15 months	2 months	6 months	Neonatal	Neonatal	Neonatal	Neonatal	6 months
Seizure type at onset	Focal	Spasms	Focal	Spasms	(Myo)clonic	Clonic	Focal	Focal	Focal
Seizure frequency at onset	Daily	Daily, multiple clusters	Daily	Daily, multiple clusters	Daily	Daily	Daily	Daily	Daily
Further seizure type(s)	Infantile spasms	Tonic-clonic, atypical absences	Myoclonic, clonic	Focal, absences, tonic, tonic-clonic	Focal	Focal, tonic	Clonic	Tonic, clonic	Clonic
AEDs trialled	Pyridoxine, vigabatrin, valproic acid, levetiracetam, clobazam, topiramate	Clonazepam, clobazam, felbamate, rufinamide, levetiracetam, valproic acid, lamotrigine	Clonazepam, topiramate, phenobarbital	Phenobarbitone, phenytoin, topiramate, levetiracetam, valproic acid, (ketogenic diet)	Phenytoin, carbamazepine, clonazepam, topiramate, levetiracetam, valproic acid, pyridoxine, vigabatrin, perampanel	Phenobarbital, phenytoin, vigabatrin, valproic acid	Phenobarbital, valproic acid, topiramate, vigabatrin, levetiracetam pyridoxine	Phenobarbital, Levetiracetam and valproic acid	Phenobarbital, levetiracetam, oxcarbazepine and topiramate
AED response	No response	Partial response to valproic acid	No response	Ketogenic diet effective (given together with phenytoin, topiramate, phenobarbitone and clonazepam)	No response	Poor response	No response	Partial response to valproic acid and levetiracetam	No response
Seizure outcome	Refractory epilepsy without any significant response to polytherapy	Refractory epilepsy	Refractory epilepsy	Refractory epilepsy	Refractory epilepsy The child died at 3 months (probable SUDEP)	Seizures every 10 to 15 days	Refractory epilepsy The child died at 5 months (probable SUDEP)	Seizures during fever	Refractory epilepsy
EEG at onset	Multifocal spikes and sharp waves with normal background during the early neonatal period	Polyspike and slow spike and wave	First EEG at 6 weeks: normal; 2nd EEG at 2 months: rare spikes and slow spikes in right centro- temporal region	First EEG at 6 months: normal; 2nd video-EEG at 7 months: focal impaired awareness seizures with left or right frontal origin	Normal during the first 7 days of life despite clinical myoclonic events	Normal at the onset	Burst-suppression like pattern	Focal discharges over the frontal and parietal areas and an abnormally slow background	Bilateral right frontal epileptic discharges

EEG at follow up	Multifocal spikes; abnormal background (slow & high voltage)	Polyspike and slow spike and wave	Multifocal spikes and slow background	EEG at 5 years: bilateral epileptiform activity (left more than right) and abnormally slow background. Episodes of intermittent eye and head deviation to right, intermittent facial jerking, abnormal movements of upper and lower limb without EEG correlate	Bilateral, mainly temporal, non-synchronized epileptic activity with multiple focal ictal electrographic events and a normal background activity in between	Very slow with fronto-central delta activity and left frontal discharges	Bilateral, mainly temporal, non-synchronized epileptic activity with multiple focal ictal electrographic events	Intermittent series of discharges of high amplitude with a maximum over the fronto-central areas (left>right).	Multifocal epileptiform abnormalities on abnormally slowed background
Last EEG	Multifocal spikes; abnormal background (slow & high voltage)	n/a	Slow background, poor organization	n/a	n/a	n/a	n/a	n/a	n/a
Central visual impairment	Yes	No	Yes	No	n/a	n/a	n/a	n/a	n/a
Photosensitivity	No	No	No	n/a	n/a	No	n/a	n/a	n/a
Other features during seizures	Oculogyric crisis	Episodes of hyperventilation	n/a	n/a	n/a	Episodes of abnormal breathing and sialorrhea	Facial grimacing, cyanosis, respiratory difficulties	Staring and blinking with eye often precede crises	Drooling, lip retraction, and gaze deviation
RTT-like features	Postnatal arrest of head growth<br>Lack of purposeful hand use	Stereotyped hand movements,<br>Abnormal breathing patterns,<br>Head nodding,<br>Inability to walk	Postnatal deceleration of head growth<br>Chronic constipation	n/a	n/a	Postnatal deceleration of head growth	n/a	n/a	n/a

Supplementary Table 7. Electro-clinical phenotype in GRIA2 NDD-EE

AED= Antiepileptic drug; EEG= Electroencephalography; RTT= Rett syndrome; SUDEP= Sudden Unexpected Death from Epilepsy

Mutant	Hom Current	Co-ex Current		HomRI	Co-ex RI		GluA1 Total	GluA2 Total	GluA1 Surface	GluA2 Surface
G47E	↓↓↓	(↓)		*nt*	↓↓↓		(=)	(=)	(=)	↓
D302G	↓↓↓	↓↓↓		*nt*	↓↓↓		(=)	(↓)	(=)	(↓)
I375V	↑	(=)		*nt*	(=)					
P528T	(=)	(=)		*nt*	(↓)		(=)	(=)	(=)	(=)
Δ528-530	↓	(↓)		*nt*	↓		(=)	(=)	(↓)	(=)
Q607E	↑	(=)		↓↓↓	↓↓↓		(=)	(↓)	(=)	↓
G609R	↓↓↓	↓↓↓		*nt*	↓↓↓		(=)	(=)	(↓)	(=)
D611N	(=)	(↓)		↑	(↓)		(↓)	(↓)	(↓)	(↓)
A639S	↓↓↓	↓↓		*nt*	↓↓		(↓)	↓↓	(↓)	↓↓
A643T	↓↓↓	*nt*		*nt*	*nt*		*nt*	*nt*	*nt*	*nt*
F644L	↓↓↓	↓↓↓		*nt*	(=)		(=)	(=)	(↓)	(=)
T646N	↓↓↓	↓		*nt*	↓		(↓)	(↓)	(=)	(↓)
V647L	(=)	(↓)		*nt*	(↓)		(=)	(↓)	(=)	(↓)

Supplementary Table 8. Summary of physiological experiments.

(↓) indicates a change was not significant after Holm-bonferroni multiple comparison adjustment, but was $p<0.1$ on the original Welch-corrected p-value

(=) indicates lack of evidence of difference from WT. We do not conclude that the mutation had no effect.

nt not tested
