# Supplementary material for: AMPA receptor GluA2 subunit defects are a cause of neurodevelopmental disorders
Source: Nat Commun. 2019 Jul 12;10:3094. doi: 10.1038/s41467-019-10910-w (PMC6626132; doi:10.1038/s41467-019-10910-w)
Supplement: Supplementary file 4 — Description of Additional Supplementary Files [file 41467_2019_10910_MOESM4_ESM.docx]

**Description of Supplementary Files**

**File Name:** **Supplementary Movie 1.**

**Description:** Follow-up video-EEG recording of the Patient 1 carrying the de-novo p.W788L variant (affecting the GluA2Flop isoform) showed high-voltage delta activity and superimposed sharp wave-slow wave complexes over the anterior cerebral regions followed by left eye deviation and tonic posturing of the upper limb (0:33) associated with right frontal slow wave-spike wave complex.

**File Name: Supplementary Movie 2.**

**Description:** Patient 1 carrying the de-novo p.W788L variant affecting the GluA2Flop isoform at the age of 3 years. Note the severe hypotonia and the oculogiric cryses.

**File Name:** **Supplementary Movie 3.**

**Description:** Patient 2 carrying the de-novo p.Pro528_Lys530del GRIA2 in-frame deletion. Note normal motor development in early infancy. At the age of 11 years, note the aggressive behaviour, the screaming and the stereotyped hand movements.

**File Name:** **Supplementary Movie 4.**

**Description:** Patient 3 carrying the de-novo p.D611N GRIA2 variant affecting the GluA2 pore. Note the screaming episodes, the stereotypies and abnormal behaviour with head banging and self-harm.

**File Name:** **Supplementary Movie 5.**

**Description:** Patient 7 carrying the de-novo p.Q607E (or p.R607G) variant affecting the GlUA2 Q/R RNA editing site. Note dystonic posturing, stereotypies and RTT-like breathing abnormalities with hyperventilation.
